# Rethinking CRITID Procedure of Brain Targeting Drug Delivery: Circulation, Blood Brain Barrier Recognition, Intracellular Transport, Diseased Cell Targeting, Internalization, and Drug Release

**DOI:** 10.1002/advs.202004025

**Published:** 2021-02-24

**Authors:** Shaobo Ruan, Yang Zhou, Xinguo Jiang, Huile Gao

**Affiliations:** ^1^ Key laboratory of Drug Targeting and Drug Delivery Systems of the Education Ministry Sichuan Engineering Laboratory for Plant‐sourced Drug and Sichuan Research Center for Drug Precision Industrial Technology West China School of Pharmacy Sichuan University Chengdu 610041 China; ^2^ Department of Pharmaceutics College of Pharmacy University of Florida Gainesville Florida 32610 USA; ^3^ Key laboratory of Smart Drug Delivery Ministry of Education School of Pharmacy Fudan University Shanghai 201203 China

**Keywords:** brain‐targeting, drug delivery, dual‐targeting, intracellular trafficking, stimulus‐responsive

## Abstract

The past decades have witnessed great progress in nanoparticle (NP)‐based brain‐targeting drug delivery systems, while their therapeutic potentials are yet to be fully exploited given that the majority of them are lost during the delivery process. Rational design of brain‐targeting drug delivery systems requires a deep understanding of the entire delivery process along with the issues that they may encounter. Herein, this review first analyzes the typical delivery process of a systemically administrated NPs‐based brain‐targeting drug delivery system and proposes a six‐step CRITID delivery cascade: circulation in systemic blood, recognizing receptor on blood‐brain barrier (BBB), intracellular transport, diseased cell targeting after entering into parenchyma, internalization by diseased cells, and finally intracellular drug release. By dissecting the entire delivery process into six steps, this review seeks to provide a deep understanding of the issues that may restrict the delivery efficiency of brain‐targeting drug delivery systems as well as the specific requirements that may guarantee minimal loss at each step. Currently developed strategies used for troubleshooting these issues are reviewed and some state‐of‐the‐art design features meeting these requirements are highlighted. The CRITID delivery cascade can serve as a guideline for designing more efficient and specific brain‐targeting drug delivery systems.

## Graphical abstract

This review first proposes a six‐step CRITID delivery cascade for dissecting the entire delivery process of brain‐targeting nanoparticle delivery systems. By reviewing the issues and requirements that limit delivery efficiency at each step, as well as corresponding strategies for troubleshooting, this review seeks to provide a guideline to design brain‐targeting drug delivery systems with improved delivery efficiency at each step.

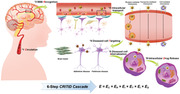

## 1 Introduction

Despite great advances in brain research, brain diseases, such as brain tumors and neurodegenerative diseases, remain the leading threats of human health.^[^ ^1^ ^]^ The current strategies available to treat these brain diseases still rely on the administration of chemical or biological drug formulation. However, the treatment outcome of many approved pharmacological cares and potential therapeutic candidates is rendered modest due to their inability to effectively cross the blood‐brain barrier (BBB) and selectively distribute to diseased cells.^[^ ^2^ ^]^ Therefore, there is a need to deliver drugs across the BBB efficiently and localize them at diseased lesion specifically with enhanced concentration.

The past decade has witnessed the explosive development of nanoparticle (NP) as drug delivery system for therapeutic or diagnostic application.^[^ ^3^ ^]^ As expected, NPs‐based drug delivery system has also been widely used for efficiently overcoming the BBB and delivering drugs specifically to brain diseased lesion or cells.^[^ ^4^ ^]^ To date, a variety of NPs with unique physicochemical properties are tailored as brain‐targeting drug delivery systems, especially when functionalized with specific targeting ligands.^[^ ^5^ ^]^ These ligand‐functionalized NPs are proved to be able to actively recognize specific receptors on BBB, leading to targeting delivery to brain and improved BBB transcytosis. Although these brain‐targeting NPs hold promise in improving therapeutic efficiency of drugs toward brain diseases, their potential have yet to be sufficiently exploited due to the limited drug accumulation in diseased cells as well as off‐target side‐effects. Therefore, to further increase the therapeutic potential of brain‐targeting NPs and reduce off‐target side‐effects, designing brain‐targeting NPs delivery system with the ability to deliver drugs to diseased cells more specifically and effectively will be greatly important.

**Figure 1:**
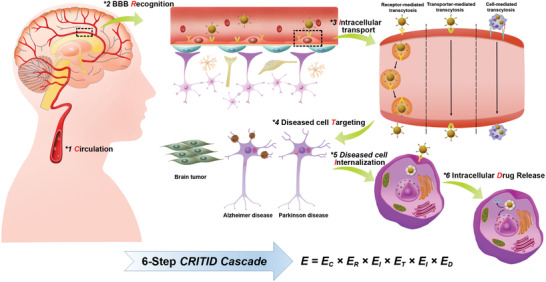
Schematic diagram of the proposed CRITID delivery cascade of brain‐targeting NPs delivery system: circulation in systemic blood, recognizing receptor on BBB, intracellular transport with BECs, diseased cell targeting after entering into parenchyma, internalization by diseased cells and finally intracellular drug release. The overall efficiency (E) is the product of six step's efficiencies.

However, rational design of brain‐targeting delivery systems should not only consider the requirements of brain targeting delivery and BBB transcytosis, but also consider their delivery fates in bloodstream, brain endothelial cells (BECs), brain parenchyma as well as diseased cells. In this review, we provide a comprehensive overview of a cascade of six critical events in the delivery process of brain‐targeting NPs: circulation in systemic blood, recognizing receptor on BBB, intracellular transport with BECs, diseased cell targeting after entering into parenchyma, internalization by diseased cells and finally intracellular drug release‐briefly, the CRITID delivery cascade (**Figure** **1**). In an effort to guarantee the maximum therapeutic efficacy, designing the brain‐targeting delivery system require a better understanding of the specific issues that limit targeting delivery efficiency and requirements at each step. However, these requirements for each step may be different or even opposite. Thus, by reviewing the currently developed strategies for troubleshooting and highlighting some state‐of‐art designs, we seek to provide a step‐by‐step guideline for designing more specific and efficient brain‐targeting delivery system.

## 2 Specific Issues and Requirements for Brain‐Targeting Drug Delivery

### 2.1 Blood Circulation

For intravenously administrated brain‐targeting NPs, they have to travel in the blood circulation before reaching brain target. Regardless of what mechanisms are involved in brain targeting, the ability of nanomedicines to accumulate at the target site is directly correlated to their blood circulation time, since longer circulation time ensures longer contact time of NPs with target tissue.^[^ ^6^ ^]^ However, the rapid opsonization‐mediated phagocytosis by mononuclear phagocyte system (MPS) along with renal excretion are the major challenges causing rapid clearance, nonspecific biodistribution, and short blood circulation time.^[^ ^7^ ^]^ Thus, understanding the interaction between biological systems and NPs is critical for controlling their blood circulation time and biodistribution.

Once entering into systemic circulation, proteins and other biomolecules rapidly compete for adsorbing onto the NP surface, leading to the formation of protein corona.^[^ ^8^ ^]^ The formation of protein corona endows them a “biological identity” and many of these proteins act as opsonins, which make them more recognizable by MPS.^[^ ^9^ ^]^ The MPS, also termed the reticuloendothelial system, is comprised of circulating dendritic cells, monocytes, and tissue‐resident macrophages of the liver, spleen, lung, and bone marrow.^[^ ^10^ ^]^ The phagocytic cells are able to effectively capture foreign objects through opsonization‐mediated phagocytosis, thus resulting in a significant portion of NPs are scavenged rapidly from blood circulation after intravenous administration.^[^ ^11^ ^]^

In addition to the MPS, the circulation time of NPs can also be affected by renal excretion, a process relies on glomerular filtration in kidney.^[^ ^12^ ^]^ The glomerular barrier is responsible for rapidly filtering circulating blood, with the properties that allow for high filtration rates of water, nonrestricted filtration of small molecules, but restricted filtration of macromolecules or proteins.^[^ ^13^ ^]^ The glomerular barrier is consisted of podocytes, glomerular basement membrane, endothelium, the endothelial cell surface layer, mesangial cells, and plasma components, which collaborate together to strictly control the filtration of substances. The glomerular filtration of circulating NPs in the bloodstream highly relies on their size and it has been proven that NPs with size less than 6 nm are able to efficiently pass through the glomerular barrier.^[^ ^14^ ^]^ Together, how to circumvent the MPS capture and renal excretion is critical to prolong blood circulation time of brain‐targeting NPs.

### 2.2 BBB Recognition

Although the etiology and pathophysiology of different brain diseases, such as brain tumor, Parkinson's disease (PD), Alzheimer's disease (AD), and ischemic stroke are not completely understood, they all share a common physiological barrier—the BBB.^[^ ^4^, ^15^ ^]^ The BBB, consisting of BECs sealed by tight junction lining the cerebral microvasculatures, and closely associated basement membrane, perivascular mast cells (pericytes), astrocytes, and smooth muscle cells.^[^ ^16^ ^]^ The nonfenestrated endothelial cells along with their intercellular tight junctions located in the luminal site of cerebral microvasculature, which present the first physical barrier to restrict the paracellular transport of large or hydrophilic molecules while only allow the passage of water, gas and certain lipid‐soluble molecules via passive diffusion.^[^ ^17^ ^]^ This physical barrier is further enhanced by pericytes and astrocytes endfeet, which is located in the abluminal site of microvasculature.^[^ ^18^ ^]^ Pericytes are multi‐functional cells embedded within the basement membrane and cover the capillary endothelium throughout the body. Astrocytes further enclose the basement membrane, with nearly 100% coverage by astrocyte endfeet. Moreover, it has also been reported that pericytes and astrocytes are also involved in regulating and maintaining BBB integrity by inducing formation of tight junction protein.^[^ ^19^ ^]^ In addition, BECs, pericytes and astrocytes are able to secrete extracellular matrix (ECM) proteins that are mainly enriched in basement membrane between astrocytic endfeet and endothelial cells.^[^ ^20^ ^]^ These ECM proteins, including lamina, fibronectin, denatured collagens (gelatin), and proteoglycans, are also vital components of the BBB and involved in regulating and maintaining BBB function.^[^ ^21^ ^]^ Given to its integrity and highly restricted permeability, BBB plays an important role in maintaining brain homeostasis by strictly controlling the substance exchange.

However, the highly restricted BBB also pose a formidable challenge to the pharmaceutical diagnosis and treatment, with more than 98% of the small molecules and nearly all large molecules such as gene and protein medicines fail to reach the brain to an appreciable extent.^[^ ^2^, ^22^ ^]^ Moreover, although the BBB endothelium expresses several kinds of influx transporters that can also be used for transporting drug with mimic structure across the BBB, it also express several efflux transporters especially the adenosine triphosphate (ATP)‐binding cassette transporters, such as P‐glycoprotein, breast cancer resistance proteins, and multidrug resistance‐associated proteins, at the luminal membrane of endothelial cells.^[^ ^23^ ^]^ These efflux transporters can further prevent drugs entering into the brain parenchyma by exporting them from endothelial cells back into circulating blood. Therefore, drugs recognized by these efflux transporters are expected to further show low distribution in the brain.^[^ ^24^ ^]^ As aforementioned, NPs have emerged as intriguing tool potentially delivering therapeutics across the BBB with improved accumulation. To date, several mechanisms have been established to assist the transport of molecules or proteins into the brain, which can also be used for mediating the transcytosis of NPs across the BBB, including receptor‐mediated transcytosis (RMT), carrier‐mediated transcytosis (CMT), and adsorptive‐mediated transcytosis (AMT).^[^ ^25^ ^]^ AMT based on the electrostatic interaction between negatively charged BBB endothelium and positively charged NPs has been widely proved to be able to mediate NPs transport across the BBB efficiently, whereas exhibiting poor selectivity.^[^ ^4^, ^25^ ^]^ By contrast, RMT and CMT represent the most common mechanisms that involved in active targeting delivery of brain‐targeting NPs, exhibiting better selectivity. Thus, endowing brain‐targeting NPs with the ability to recognize receptors or transporters on the BBB is a fundamental prerequisite to ensure selective brain‐targeting delivery and effective BBB transcytosis.

### 2.3 Intracellular Transport

Although these aforementioned mechanisms are now being widely used for assisting entrance of NPs into brain, they can be quite distinct and are still not well understood. For the RMT, the mechanism used for crossing the BBB mainly involves three steps, including endocytosis, intracellular vesicle trafficking and exocytosis. Briefly, once ligand‐modified NPs from the peripheral blood bind to specific receptor on luminal (apical) site of BECs membrane, the endocytosis process is initiated. The endocytosis is accompanied by invagination of the cellular membrane around the ligand‐receptor complex cluster and wrapping of this cluster, leading to formation of a membrane‐bound intracellular transport vesicle. The cargo‐loaded vesicle then undergoes an intracellular trafficking throughout the cytoplasm to the opposing (basolatoral) site of BECs membrane, where it fuses with membrane. Subsequently, the ligand is dissociated from receptor to release the cargo into brain parenchyma while the receptor is recycled back to either apical membrane or lysosome.^[^ ^26^ ^]^ Apart from transcytosis, the vesicle trafficking can also be subject to other fates due to specific sorting pathway, such as targeting to an intracellular organelle (e.g. Golgi apparatus), routing back to the apical membrane from early endosome or recycling endosome, or fusing with lysosomes for cargo degradation.^[^ ^27^ ^]^ It was reported that high‐affinity anti‐transferrin (Tf) antibodies bound to the transferrin receptor (TfR) remained firm association with TfR and eventually traffic to lysosomes, leading to the “trapping” in the BECs and insufficient transcytosis.^[^ ^28^ ^]^ Moreover, studies showed the lower lysosomal colocalization while better transcytosis of monovalent anti‐TfR antibody than divalent anti‐TfR antibody.^[^ ^29^ ^]^ These findings suggested that the affinity of antibody may play an important role in determining the intracellular trafficking fate. Similarly, the intracellular trafficking of most developed brain‐targeting NPs based on RMT may also face the same issue and is often overlooked in the entire delivery processes. Following study confirmed the hypothesis that high‐affinity ligand‐functionalized NPs were more likely to be trapped within lysosome of BECs, resulting in reduced transcytosis.^[^ ^30^ ^]^ Therefore, how to circumvent the vesicle trafficking of brain‐targeting NPs to other destination especially lysosome or minimize the potential to be trapped in BECs will be greatly important for increasing the transcytosis brain parenchyma.

### 2.4 Diseased Cell Targeting

The ability of brain‐targeting NPs to distinguish between diseased cells and normal cells after entering brain parenchyma remains to be a challenge. If the NPs are unable to specifically target the diseased cells, and instead, nonspecifically distribute in the whole brain, the treatment outcome will be subject to the limited concentration of drug. Increasing the drug payload can potentially improve the therapeutic effect, while normal tissue has to suffer from even worse side‐effect due to the nonspecific distribution of drug. Therefore, an ideal brain targeting drug delivery system should possess the capacity to not only target the BBB but also selectively target diseased cell after entering into brain parenchyma.^[^ ^31^ ^]^ However, the targets of different brain diseases may be different, thus targeting diseased cells with high specificity requires a deep understanding of the pathogenic and pathological characteristics of different brain diseases.

Brain tumors are a diverse group of primary and metastatic neoplasms in the central nervous system (CNS), including diffusely infiltrating glioma, anaplastic astrocytomas, glioma, and brain metastasis (BM), continue to be the cause of disproportionate morbidity and mortality.^[^ ^32^ ^]^ Among these, glioma is the most common malignant brain tumors characterized by highly proliferative, aggressive, and distinctive pathological heterogeneities, accounting for 15% of all brain tumors.^[^ ^33^ ^]^ It has been widely reported that the progression of glioma cells is associated with a variety of overexpressed receptors, including TfR, low‐density lipoprotein receptor (LDLR), LDLR‐related protein (LRP), integrin receptor, epidermal growth factor receptor (EGFR), and glucose transporter (GLUT), which can serve as potential targets for developing glioma‐targeting NPs.^[^ ^34^ ^]^

AD is a progressive neurodegenerative disorder and the most common form of dementia. The two main pathological hallmarks of AD are the deposition of extracellular plaques in the cerebral neuropil and intraneuronal neurofibrillary tangles (NFTs).^[^ ^35^ ^]^ Amyloid‐*β* (A*β*) peptide and phosphorylated tau protein (p‐tau) are the primary components of the plaques and tangles, respectively. A*β* peptides are derived from amyloid beta precursor protein (APP) peptides through the sequential proteolytic processing by the *β*‐site APP cleavage enzyme 1 (BACE1) and *γ*‐secretase, forming A*β* fragments with varying number of amino acids ranging from 36–42.^[^ ^36^ ^]^ By contrast, tau is a critical neuronal microtubule‐stabilizing protein that contributes to microtubule assembly and axonal outgrowth.^[^ ^37^ ^]^ Evidence has confirmed that the accumulation of upregulated hyperphosphorylation and other post‐translational modifications could convert tau from a multifunctional protein to a neurotoxic entity, triggering tau oligomerization/aggregation and resulting in overtime NFTs and ultimately irreversible neurodegeneration.^[^ ^38^ ^]^ Therefore, NP‐based diagnostic and therapeutic strategies that target A*β*‐induced neurotoxicity and/or tau‐induced neurotoxicity are an attractive approach to treat AD.^[^ ^39^ ^]^

PD is another prevalent neurodegenerative disorder particularly characterized by the loss of dopaminergic neurons in the substantia nigra pars compacta that cause complex and variable motor and nonmotor symptoms.^[^ ^40^ ^]^ Deposition of protein aggregation containing cytoplasmic *α*‐synuclein (termed lewy bodies) in multiple dopaminergic neurons is one of the major hallmarks of advanced PD patients.^[^ ^41^ ^]^ Although the etiology of PD has not yet been fully understood, a variety of underlying mechanisms are believed to be involved in the pathogenesis of PD, including mitochondrial dysfunction, oxidative stress, and neuroinflammation.^[^ ^42^ ^]^ Therefore, these pathological heterogeneities can serve as therapeutic targets for developing potential therapies for PD. Moreover, dopaminergic neurons from PD brain also possess several receptors with upregulated expression, such as dopamine transporter (DAT),^[^ ^43^ ^]^ and nicotinic acetylcholine receptors (nAchR).^[^ ^44^ ^]^

Stroke is a leading cause of adult long‐term disability and death worldwide.^[^ ^45^ ^]^ Ischemic stroke, accounting for ≈90% of all stroke cases, is induced by the formation of cerebrovascular embolism or thrombosis that blocks the supply of oxygen and nutrients to neurocytes, followed by the damage of brain tissue caused by hypoxia, free radicals, and inflammation.^[^ ^46^ ^]^ Moreover, accumulating evidence suggest that reperfusion following thrombolytic therapy can still induce neuroinflammation and free radicals’ production due to the oxygen‐boost in the ischemic site, resulting in secondary damage to neurons.^[^ ^47^ ^]^ Thus, inflammation and free radicals are currently considered the major therapeutic targets for the development of new stroke therapies. Similarly, neurons from stroke brain also exhibit upregulated expression of nAchR, offering the opportunities to design stroke‐targeting NPs.

### 2.5 Diseased Cell Internalization

For those therapeutic agents whose targets are localized in the intracellular compartments of diseased cell, efficient translocation across the cell membrane to subcellular compartments is thereby an important prerequisite for exerting therapeutic effect.^[^ ^48^ ^]^ Dependent on the physiochemical characteristics of NPs and the nature of target cells, cellular internalization of NPs may involve active transport (either the phagocytosis or the endocytosis) and passive transport (direct penetration).^[^ ^49^ ^]^ As described above, phagocytosis primarily occurs when professional phagocytes encounter NPs, especially for those with size larger than 500 nm.^[^ ^50^ ^]^ In contrast, endocytosis occurs in virtually all cells, including clathrin‐mediated endocytosis, receptor‐mediated endocytosis (RME), caveolae‐mediated endocytosis, micropinocytosis, and other endocytosis pathway independent of clathrin and caveolae.^[^ ^49a^ ^]^ Among these, RME is considered as the major mechanism involving in the internalization of ligand‐functionalized NPs.^[^ ^49^, ^51^ ^]^ To further optimize the internalization of brain‐targeting NPs by diseased cells, understanding the factors that affect the efficiency of RME will be greatly important.

### 2.6 Intracellular Drug Release

In order to elicit therapeutic function, many drugs, including small molecule anticancer drugs, proteins, siRNA, and DNA, have to be released in the form of free drugs into specific subcellular compartments, typically the cytoplasm or nucleus of diseased cell after internalization. Although ligand‐functionalized NPs can significantly enhance cellular uptake by diseased cell, insufficient drug concentration still occurs due to a variety of barriers including poor drug release, membrane‐associated multidrug‐resistance mechanism,^[^ ^52^ ^]^ and delivery resistance toward subcellular compartments.^[^ ^53^ ^]^ Generally, conventional NP‐based formulations tend to passively release drug payloads via diffusion‐controlled release pathway after internalization, leading to insufficient drug concentration at the desired time window.^[^ ^54^ ^]^ In addition, the internalized ligand‐modified NPs via RME usually undergo a vesicle trafficking to endosomes or lysosomes,^[^ ^49a^ ^]^ wherein the acidic pH and various proteolytic enzyme deactivate the released drugs if their therapeutic target is cytoplasm, mitochondria, or nucleus.^[^ ^55^ ^]^ Therefore, rapid and sufficient drug release with the ability to escape from lysosome (if necessary) is highly required following internalization into diseased cells.

## 3 Strategies for Troubleshooting

### 3.1 Blood Circulation

A widely accepted idea to increase blood circulation time of brain‐targeting NPs is tailor their physicochemical properties (such as size, shape, and surface charge), which play an important role in determining opsonization‐directed MPS phagocytosis and renal excretion.^[^ ^56^ ^]^ Additionally, surface modification such as PEGylation^[^ ^57^ ^]^ or biomimetic camouflaging (self‐marker CD47‐functionalization,^[^ ^58^ ^]^ cell‐membrane coating^[^ ^59^ ^]^) has emerged as promising strategy for reducing nonspecific capture and thus prolonging the blood circulation time of NPs.

#### 3.1.1 Tailoring Physicochemical Properties

Particle size plays an important role in determining the in vivo biodistribution and circulation time. It is generally considered that NPs with a size of less than 10 nm undergo rapid renal excretion from blood circulation upon intravenous injection, unless they are prepared using degradable materials such as polymers, lipid, or hydrogels.^[^ ^7^, ^60^ ^]^ For example, Choi et al. revealed that quantum dots with a final hydrodynamic diameter less than 5.5 nm were rapidly eliminated through renal excretion.^[^ ^61^ ^]^ For the NPs that are large enough to avoid renal excretion, the non‐specific MPS phagocytosis contributes to their major clearance. It has been demonstrated that particles with size larger than 200 nm can activate the human complement system and can be quickly removed from blood, accumulating in the liver and spleen where they are phagocytosed by the MPS cells.^[^ ^60^ ^]^ Study demonstrated that polymeric NPs with a size less than 200 nm had an increased blood circulation time, likely due to small‐size NPs possessed a surface with small radius of curvature preventing the adsorption of opsonin.^[^ ^62^ ^]^ In an effort to obtain long‐circulating NPs, the size of particles should be large enough to avoid renal filtration but small enough to minimize MPS clearance. Based on the current data, spherical particles with a size range of 10–200 nm seems to be optimal, while the presumed threshold sizes need more confirmation.

The blood circulation time of NPs is also highly dependent on their shape.^[^ ^63^ ^]^ It has been revealed that the local geometrical parameters of NPs at the point of first contact with cells, such as curvature and aspect ratio, determine the propensity to be internalized by macrophage or simply attached.^[^ ^64^ ^]^ NPs with a length of normalized curvature, denoted as Ω, less than or equal to 45° can be internalized successfully via actin‐cup and ring formation. By contrast, NPs with a Ω ≥ 45° can be attached on macrophages, whereas exhibit near‐complete resistance to phagocytosis. For example, Geng et al. have demonstrated that filamentous polymer micelles (filomicelles) have much longer circulating times (> 1 week after administration) than spherical counterparts (2–3 days).^[^ ^65^ ^]^ Thus, in order to evade MPS capture, particles with ellipsoidal, cylindrical, and discoidal shape featured by high aspect ratios and low regions of curvature are highly preferred, such as worm‐like NPs^[^ ^66^ ^]^ and filomicelles.^[^ ^64b^ ^]^ It should be noted that the understanding of the shape effect on blood circulation is still limited due to the lack of proper synthetic methods to prepare uniform NPs with a finely tuned morphology.^[^ ^57^ ^]^

Surface charge can also significantly influence the blood circulation time. Positively charged NPs tend to absorb and aggregate with serum proteins, leading to increased MPS phagocytosis and rapid clearance rate. In contrast, neutral or negatively charged NPs are less likely to absorb serum proteins and thus show lower MPS phagocytosis, contributing to long circulation time.^[^ ^67^ ^]^ For example, Yamamoto et al. demonstrated the advantage of neutral and negatively charged polymeric micelles for long circulation time and showed negatively charged NPs significantly reduced the non‐specific uptake by liver and spleen.^[^ ^68^ ^]^ Theoretically, NPs with a slightly negative surface charge are ideal for minimizing non‐specific uptake by MPS and prolonging blood circulating time. However, in some case, neutral and positively charged liposomes show reduced MPS uptake and are cleared slower than negatively charged ones, presumably due to that negatively charged liposomes can potentially bind to available cationic sites on the macrophage surface and be recognized by scavenger receptor.^[^ ^69^ ^]^ The inconsistent results from the above studies may be explained by the different composition of NPs.

#### 3.1.2 Surface Modification

Modification of the surface properties of NPs is the most widely used strategy to prevent NPs from clearance and further prolong their blood circulation time.^[^ ^6a^ ^]^ The general objective of surface modification is to decrease NP hydrophobicity and surface charge density, thus preventing them from opsonization and allowing for the escape from the MPS.^[^ ^6a^ ^]^ It has been well‐documented that camouflaging NPs with antibiofouling synthetic polymers or cell membrane represents the most promising strategy to improve blood circulation time.^[^ ^57^ ^]^

Poly(ethylene glycol) (PEG) is the most common polymer used for grafting onto NPs’ surface to endow them with stealth functionality, due to its high hydrophilicity, chain flexibility, electronic neutrality, and absence of functional groups, a process called PEGylation.^[^ ^70^ ^]^ The PEGylation strategy has demonstrated great success in effectively reducing the MPS uptake and prolonging blood circulation time, due to the ability of PEG units to form a steric hydrated “cloud” layer on the surface of NPs. This hydrated layer in turn shields protein‐binding sites and prevents opsonin absorption, making NPs invisible to MPS.^[^ ^57^, ^67^ ^]^ For example, the circulation time of PEGylated doxorubicin (DOX)‐liposome increased from minutes to hours compared with unmodified ones.^[^ ^71^ ^]^ Moreover, studies have revealed that not only the overall PEG length and grafting density,^[^ ^72^ ^]^ but also the configuration and conformation affect the protein‐resistant ability of PEG.^[^ ^67c^ ^]^ It is generally thought that a higher PEG density can significantly prolong circulation time, while it may somehow affect the targeting efficiency^[^ ^73^ ^]^ as well as the interaction with disease cell.^[^ ^74^ ^]^ Moreover, recent observation of anti‐PEG immunological responses has triggered the exploration of alternative options for stealth coating.^[^ ^75^ ^]^ Zwitterionic materials such as poly(carboxybetaine) have been proposed as promising alternatives, which are highly resistant to protein absorption because of their strong hydration.^[^ ^76^ ^]^

CD47, an integrin‐associated transmembrane glycoprotein, is found expressed on all cell membrane in humans, mice, and other mammals. One of the critical roles of CD47 is acting as “marker of self” that up‐regulated on hematopoietic cells, leukemia cells as well as multiple cancer cells to evade macrophages.^[^ ^77^ ^]^ The interaction of CD47's extracellular domain with the CD172a (also known as signal regulatory protein‐*α*, SIRP*α*) on macrophage activate the “don't‐eat‐me” signal transduction pathway, which blocks the phagocytosis of “self” cells by macrophage.^[^ ^77a^ ^]^ Inspired by this self‐protection, emerging studies using recombinant protein CD47 or short‐chain CD47‐mimicking peptides to functionalize NPs have proved the practicality of blocking MPS uptake. Discher et al. have explored an active camouflaging strategy that utilized the CD47 “self” peptides to modify onto the surface of NPs for reducing phagocytosis.^[^ ^58^ ^]^ The circulation time of CD47 “self” peptide‐coated NPs was significantly prolonged after intravenous administration into mice. Therefore, active stealth strategy by functionalizing NPs with CD47‐mimicking peptides can be a good option to design long‐circulating NPs.

Recent advances in molecular and cellular biology have inspired the exploration of cell membrane camouflaging technique.^[^ ^78^ ^]^ In particular, camouflaging NPs with cell membranes, isolated from autologous red blood cells (RBCs) and leukocytes, provides a biomimetic surface shown to escape MPS recognition and substantially prolong in vivo circulation time. For example, RBCs membrane has been used to coat NPs to significantly reduce the macrophage phagocytosis and increase blood circulation time due to their intrinsic “self‐recognition” nature.^[^ ^79^ ^]^ Parodi et al. camouflaged nanoporous silicon particles with leukocyte membrane and showed that the resulting leuko‐like vehicles were able to efficiently avoid opsonization, delay MPS uptake and prolong circulation time.^[^ ^59^ ^]^ In addition to RBCs and leukocyte membrane, many other types of membranes have also been widely explored, including membranes from platelets,^[^ ^80^ ^]^ macrophage,^[^ ^81^ ^]^ cancer cells,^[^ ^82^ ^]^ and stem cells.^[^ ^83^ ^]^ Thus, by taking advantage of the excellent biocompatibility, intrinsic nature, and highly tunable functionality of source cell membrane, cell membrane‐camouflaged NPs hold great promise to not only prolong the blood circulation but also realize diseased cell‐specific targeting.

### 3.2 BBB Recognition

Various endogenous receptors and transporters that control the transport of molecules across the BBB endothelium have been well identified, offering the opportunities to serve as potential targets.^[^ ^84^ ^]^ The use of endogenous receptors or transporters for designing active brain‐targeting NPs can be pursued by either functionalizing with targeting ligand (often referring to substrate, substrate analogue, and antibody) or coating with specific cell membrane. These ligand‐functionalized or cell membrane‐coated NPs are able to recognize specific receptors and/or transporters expressed on the BECs.^[^ ^84^ ^]^ Herein, we highlight some of the widely used endogenous receptors/transporters as well as their specific ligands that have been identified. The potential of cell‐based recognition pattern to improve brain‐targeting delivery of therapeutics will also be discussed.

#### 3.2.1 Recognizing the Receptors on the BBB

RMT is the main mechanism involved in the transport of molecules, such as hormones or high molecular mass protein (Tf, insulin, leptin, low density lipoproteins), across the BBB.^[^ ^85^ ^]^ RMT relies on the presence of specific receptors on the BECs, including LDLR, TfR, nAchRs, scavenger receptor class B type I, insulin receptor, lactoferrin receptor, and leptin receptor.^[^ ^86^ ^]^ NPs can thus be functionalized with different types of ligands or antibodies that can recognize specific receptors and trigger subsequent transcytosis machinery to cross the BBB. Considering the need to further recognize diseased cells following transcytosis across the BBB (see detail in Diseased cell targeting section), the targets selected for BBB recognition should be more correlated with the diseases. As described above, brain tumor is also characterized by the overexpression of LDLR and TfR on cell surface while neurodegenerative disorder is characterized by the up‐regulation of nAchRs on diseased neuron cells. Moreover, the selection of potential targets should also exclude those with high expression on peripheral system. Therefore, we highlight some of the most widely used targets with high targeting specificity as well as their specific ligands and antibodies.

LDLR, a transmembrane glycoprotein, is highly expressed on BBB endothelium and mediates the uptake of cholesterol‐rich LDL, such as cholesterol, tocopherol, and Apos, into the brain.^[^ ^87^ ^]^ It has been widely proved that LDLR also plays an important role in regulating the deposition of A*β* plaques in the brain.^[^ ^88^ ^]^ The LDLR family consists of a large class of cell surface receptors, including LDLR, LRP‐1, LDL receptor‐related protein 1B (LRP1B), megalin/LRP2, very‐LDLR (VLDLR), apolipoprotein E receptor 2 (ApoER2)/LRP8, sortilin‐related receptor (SorLA/LR11), LRP5, and LRP6.^[^ ^89^ ^]^ Among these, LRP regulates the internalization of a variety of substrates, including Apos,^[^ ^90^ ^]^ P97 (melanotransferrin),^[^ ^91^ ^]^ and Angiopeps.^[^ ^92^ ^]^ Angiopeps, a family of 19 amino acid peptides derived from the kunitz domain, has emerged as a promising peptide used for targeting BBB by recognizing LRP‐1.^[^ ^93^ ^]^ Angiopep‐2 (TFFYGGSRGKRNNFKTEEY, MW 2.4 kDa) is one of angiopep family and has been widely used for assisting targeting delivery of NPs to brain disease.^[^ ^92^, ^93^ ^]^

TfR, a dimeric transmembrane glycoprotein, plays an important role in regulating transport of iron‐bound Tf across the BBB.^[^ ^94^ ^]^ Therefore, brain‐targeting NPs can be achieved by functionalizing with endogenous ligand Tf that specifically recognizes the TfR on the BBB.^[^ ^95^ ^]^ Our group has developed Tf‐coating dendrigraft poly‐L‐lysine (DGL) loaded with DOX, which showed the capacity to recognize TfR and to improve targeting efficiency compared to unmodified one.^[^ ^96^ ^]^ Despite of improved brain‐targeting efficiency, the presence of free endogenous Tf in the blood circulation may compete to bind TfR and thus restricts the overall binding efficiency of Tf‐functionalized NPs.^[^ ^97^ ^]^ To troubleshoot this issue, the use of monoclonal antibodies (OX26,^[^ ^98^ ^]^ 8D3,^[^ ^99^ ^]^ and R17217^[^ ^100^ ^]^) directly against TfR on the BBB has been developed as a promising strategy since they can recognize a distinct TfR epitope, while still granting the efficient BBB transcytosis. Additionally, peptide‐based recognition has also been proposed as a good option, such as B6 peptide (a transferrin substitute, CGHKAKGPRK)^[^ ^101^ ^]^ and T7 (HAIYPRH).^[^ ^102^ ^]^

nAchR, an ion‐gated channel receptor, represents another promising candidate due to its expression on both BECs and diseased neuron cells from neurondegenerative diseases, such as AD, PD, and stroke.^[^ ^103^ ^]^ CDX peptide (FKESWREARGTRIERG) derived from the loop II region of snake neurotoxin candoxin demonstrated high binding affinity with nAchRs.^[^ ^104^ ^]^ In addition, studies have revealed that nAchR was involved in mediating the transport of the rabies virus glycoprotein (RVG, 505 amino acid) into the neuron cells.^[^ ^105^ ^]^ Peptides derived from RVG, such as RVG‐29, are thus proposed a targeting ligands used for recognizing nAchR and mediating brain‐targeting delivery of NPs.^[^ ^106^ ^]^ Jiang et al. developed RVG29‐functionalized DGL loaded with caspase3 shRNA‐coding plasmid (RVG29‐DGL‐shRNA) for PD treatment and showed that this delivery system could reduce activated caspase‐3 levels as much as threefold in PD‐bearing rat after intravenous injection compared with unmodified control.^[^ ^107^ ^]^

#### 3.2.2 Recognizing the Transporter on the BBB

CMT is responsible for bi‐directional transport of small molecule substances, including glucose, amino acid, nucleosides, and monocarboxylic acid, across the BBB.^[^ ^5^, ^108^ ^]^ Currently, CMT has also been widely used for mediating brain‐targeting delivery and BBB transcytosis.^[^ ^109^ ^]^ Several nutrient transporter systems have been identified, with each transporting a group of nutrients of similar structure, including GLUT, large neutral amino acid transporter, cationic amino acid transporter member 1, monocarboxylic acid transporter, concentrative nucleoside transporter, choline transporter, and nucleobase transporter. Molecules that closely mimic the structure of transporter substrate are now being regarded as a potential ligand to functionalize NPs.

GLUT is located on both luminal and basolateral side of the BECs, mediating the passage of glucose and substances with similar structures through the BBB, including 2‐deoxyglucose, galactose, mannose, and glucose analogs. Considering the transporting efficiency of GLUT is significantly higher (1420 nmol min^−1^ g^−1^ tissue) than other nutrient transporters (91, 28, and 11.3 nmol ^−1^min^−1^ g tissue) for monocarboxylic acid, neutral amino acid and amine, it holds great promise as a target for designing brain‐targeting NPs.^[^ ^110^ ^]^ Peng et al. used P‐aminophenyl‐*α*‐D‐mannopyranoside (MAN), a mannose analog, to conjugate onto liposomes to enable specific recognition to GLUT1 and subsequent BBB transcytosis.^[^ ^111^ ^]^ In vivo optical imaging demonstrated that MAN‐liposome had significantly higher brain fluorescence intensity than that of unmodified liposome. One factor to taken into consideration is that since these endogenous carriers are typically small, stereospecific pores, they are not particularly amenable to the transport of large‐molecule therapeutics. However, it should be noted that specific targets utilized to enhance the drug delivery can potentially be detrimental for the normal physiology of the host. For example, utilizing GLUT for brain‐targeting delivery may cause competition to the essential glucose transport to the brain. Therefore, the exploration and utilization of potential target for developing brain‐targeting NPs should be careful and well‐balanced to not occupy all the targets. Meanwhile, additional physiological parameters should also be evaluated to assess the design and development.

#### 3.2.3 Cell‐Based Recognition Pattern

Neutrophils (NEs), a type of polymorphonuclear leukocyte, play a critical role in immune response.^[^ ^112^ ^]^ NEs have now been widely studied in cancer treatment owing to their intrinsic ability to traverse BBB/blood brain‐tumor barrier (BBTB) and penetrate the tumor site in response to the inflammation.^[^ ^113^ ^]^ Typically, the blood‐to‐brain trafficking of NEs is a multiple‐step process involving rolling, adhesion, intracellular crawling, and transmigration (paracellular pathway or transcellular pathway).^[^ ^114^ ^]^ Regardless of paracellular transmigration or transcellular transmigration, their initial recruitment shares the similar process of activation of intercellular crosstalk between NEs and endothelial cells, activation of signaling networks and specific molecules recognition.^[^ ^115^ ^]^ These specific recognition patterns mainly include selectin, intercellular adhesion molecule and integrin, which make NE a promising brain‐targeting delivery platform.^[^ ^116^ ^]^ Xue et al. developed biomimetic NEs carrying paclitaxel (PTX)‐loaded liposomes (PTX‐CL/NEs) to specifically deliver PTX to inflamed brain tissue for suppressing postoperative glioma recurrence.^[^ ^117^ ^]^ Surgical tumor resection can lead to an inflammatory reaction occurs in the brain, accompanied by the release of inflammatory factors into the blood. After intravenous administration, PTX‐CL/NEs were primed by the chemoattractants and migrated along the chemotactic gradient toward the infiltrating tumor cells in the inflamed brain. The increased inflammatory factors recruited and excessively activated the NEs, leading to the release of PTX from PTX‐CL/NEs. In vivo studies showed that PTX‐CL/NEs could greatly increase the PTX accumulation in the inflamed brain compared to PTX‐CL. After treatment with PTX‐CL/NEs, G422‐bearing mice pre‐treated with tumor resection demonstrated much prolonged median survival (61 days) compared to that treated with Taxol (29 days) and PTX‐CL (38 days) even at a double PTX dosage.

In addition to NEs, cancer cells, including lung cancer, breast cancer, and melanoma, also possess the ability to cross the BBB into brain, a process called BM.^[^ ^118^ ^]^ Approximately 20–30% of these malignant tumor cells can metastasize into brain to induce secondary brain tumors.^[^ ^119^ ^]^ The adhesion of these cancer cells to BECs and subsequent transendothelial migration also require specific recognition patterns and intercellular interactions between cell membrane receptors and endothelial cells adhesion molecules, such as integrins, selectins, and chemokines.^[^ ^120^ ^]^ Inspired by this, camouflaging NPs with cancer cell membrane may enable not only BBB targeting delivery and transcytosis but also tumor targeting delivery without the help of ligand. For example, Wang et al. developed a brain metastatic tumor cell membrane‐coated NPs with core‐shell structure to cross BBB for diagnosis and photothermal therapy of early glioma.^[^ ^121^ ^]^ In vitro and in vivo studies suggested B16F10 cell membrane (melanoma) and 4T1 cell membrane (breast cancer)‐coated indocyanine green (ICG)‐loaded NPs (B16‐PCL‐ICG or 4T1‐PCL‐ICG) could efficiently cross the BBB and entered into brain. More importantly, treatment with B16‐PCL‐ICG plus 808 nm laser irradiation led to significant glioma regression compared to COS7 (a normal cell line) membrane‐coated ICG‐loaded NPs. The results indicated the potential of cancer cell membrane‐camouflaged in mediating NPs transcytosis across the BBB and targeting delivery to glioma. Moreover, it should be noted that cancer cell membrane‐camouflaged NPs also possess the homotypic targeting ability, which is primarily dependent on the interaction between natural membrane proteins and cognate target cells.^[^ ^122^ ^]^ Jia et al designed biomimetic glioma membrane protein camouflaged liposome loaded with ICG (BLIPO‐ICG) for targeted photothermal therapy of orthotopic glioma.^[^ ^123^ ^]^ In vivo studies suggested the profound advantages of glioma cell membrane protein camouflaged NPs in prolonging blood circulation and glioma‐targeting delivery. After treatment with BLIPO‐ICG with 808 nm laser irradiation, the glioma growth was significantly inhibited compared to that treated with non‐camouflaged LIPO‐ICG with laser irradiation.

Genetically engineering T cells to express synthetic antigen receptors, based on either natural T‐cell antigen receptors or antibodies fused to T‐cell signaling domains as chimeric antigen receptor (CAR), has emerged as another promising therapeutic tool to target several types of cancer cells.^[^ ^124^ ^]^ Encouragingly, the use of CAR T for targeted treatment of brain tumors has also been explored and exhibited signs of promise with variety preclinical and ongoing clinical trials, which mainly focus on targeting the interleukin 13 receptor α2 (IL13R*α*2), human epidermal growth factor receptor 2 (HER2), and EGFR vIII receptor.^[^ ^125^ ^]^ Similar to leukocytes, the blood‐to‐brain trafficking of T cells also involves adhesion and transmigration, which is regulated by the sequential intercellular interactions between T cells and BECs. Several specific molecules are involved in the intercellular interactions, including adhesion molecules, integrin, and selectin.^[^ ^126^ ^]^ Nellan et al. developed HER2‐CAR T cells containing the 4D5 single chain variable fragment (scFv) specific for HER2, CD3 zeta signaling domain and 4‐1BB costimulatory motif, referring to as HER2‐BBz‐CAR T cells, to combat medulloblastoma.^[^ ^127^ ^]^ They found that intravenous administration of HER2‐BBz‐CAR T cells at high cell dose (2.5 × 10^6^) could produce a complete regression of medulloblastoma cells in mice. In contrast, administration of CD19‐BBz‐CAR T cells at same dose showed no significant effect on tumor growth. These results suggested the potential of HER2‐BBz‐CAR T cells to cross BBB efficiently and target medulloblastoma cells after systemic administration.

### 3.3 Intracellular Transport

As discussed above, the affinity of ligand plays a critical role in determining the intracellular trafficking fate as well as transcytosis efficiency.^[^ ^28^ ^]^ Therefore, optimizing the avidity of brain‐targeting NPs toward receptors on the BBB to minimize their possibility to be trapped within BECs represents the most basic approach.^[^ ^128^ ^]^ In addition, several other strategies such as utilizing antibody with pH‐dependent affinity,^[^ ^129^ ^]^ intracellular stimulus‐responsive detachment of NPs from ligand‐receptor complexes,^[^ ^96^ ^]^ and cell‐penetrating peptide^[^ ^130^ ^]^ have also been developed to accelerate the intracellular transport transcytosis across the BBB.

#### 3.3.1 Optimizing Avidity of Brain‐Targeting NP

**Figure 2:**
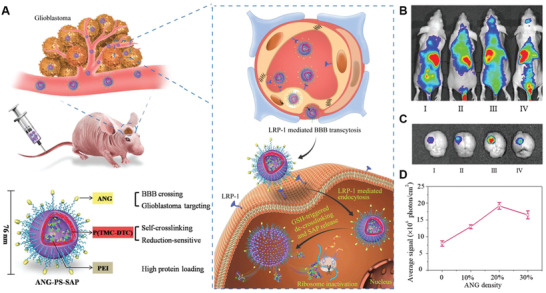
Brain‐targeting NPs with optimized ligand density. A) Schematic diagram of structure and composition of ANG‐PS‐SAP as well as proposed mechanism of glioma cell targeting after intravenous injection. B) In vivo fluorescence imaging of U87 MG‐luc glioma‐bearing nude mice at 24 h post‐injection of DiR‐loaded PS (I) and ANG‐PS with varying ANG densities (II: 10%, III: 20%, IV: 30%). C) Ex vivo fluorescence imaging of excised brains. D) Semiquantitative analysis of the fluorescence intensity of DiR‐loaded PS and ANG‐PS. Reproduced with permission.^[^ ^131^ ^]^ Copyright 2018, Wiley‐VCH.

As discussed above, reducing the affinity of anti‐TfR antibodies to TfR showed actually increased transcytosis across the BBB when blood antibody concentration was high, which was probably due to easier dissociation between low‐affinity anti‐TfR antibodies with TfR.^[^ ^28^ ^]^ Low‐affinity binding results in excellent dissociation, whereas poor initial binding. Moreover, NPs binding affinity is more complex than antibodies because NPs binding event can involve multiple receptor‐ligand interactions. Therefore, optimizing the avidity of ligand‐modified NPs by either choosing ligand with intermediate affinity or appropriate ligand density may hold promise to enhance the transcytosis of NPs. Wiley et al. reported that gold nanoparticles (AuNPs) conjugated with high concentrations of Tf (100–200 molecules of Tf per NP) stay bound to BECs. In contrast, AuNPs conjugated with low concentrations of Tf (20–30 molecules of transferrin per NP) can interact effectively with the receptor, while still undergo transcytosis and be released into brain parenchyma.^[^ ^30^ ^]^ In a recent study, Jiang et al. developed angiopep‐2 directed and redox‐responsive virus‐mimicking polymersome loaded with saporin (SAP) (ANG‐PS‐SAP) for targeted glioma therapy (**Figure** **2**A).^[^ ^131^ ^]^ By screening the angiopep‐2 density, they found that ANG‐PS with 20% density of angiopep‐2 possessed both the highest in vivo and ex vivo distribution at glioma site (Figure 2B,C). Quantitative analysis of fluorescence intensity further confirmed the finding that 20% density of angiopep‐2 modification was optimal for mediating NPs transport across the BBB (Figure 2D). The results further validated the importance of ligand density in improving intracellular transport and thus improving the amounts of NPs entering into brain.

#### 3.3.2 Cleavable Linker

Although ligand‐modified NPs with reduced avidity to receptor are less likely to be trapped within endothelial cells, they may require high quantities in the blood to guarantee sufficient amount of drug reaching the brain parenchyma.^[^ ^28^ ^]^ Therefore, how to develop NP with high affinity while still grant the ability to circumvent intracellular trapping will be greatly important. Hadassah et al. proposed anti‐TfR antibodies with pH‐dependent affinity toward TfR (high TfR affinity at neutral extracellular pH while low TfR affinity at acidic endosomes) and demonstrated that these they could efficiently cross in vitro BBB model. In contrast, antibodies with pH‐independent affinity for TfR were more likely to alter intracellular trafficking away from transcytosis toward lysosomes for degradation.^[^ ^129^ ^]^ Later on, strategies utilizing stimulus‐responsive cleavable linker to enable the detachment of NPs from high‐affinity ligand have gained increasing attention. Davis et al. developed Tf‐coating 80 nm AuNPs with an acid‐cleavable diamino ketal (DAK) linker between Tf and the NPs core.^[^ ^128^ ^]^ The DAK linker could be cleaved in response to acidic pH condition in lysosomes, enabling the detachment of AuNPs from the multidentate Tf‐TfR complex within endothelial cells. In vitro and in vivo studies showed increased ability of the Tf‐coating NPs with acid‐cleavable linker to cross the BBB, and more importantly, entering into brain parenchyma in greater amount. However, the detached NPs also had the propensity to recycle back into peripheral blood instead of entering into brain parenchyma.^[^ ^132^ ^]^

**Figure 3:**
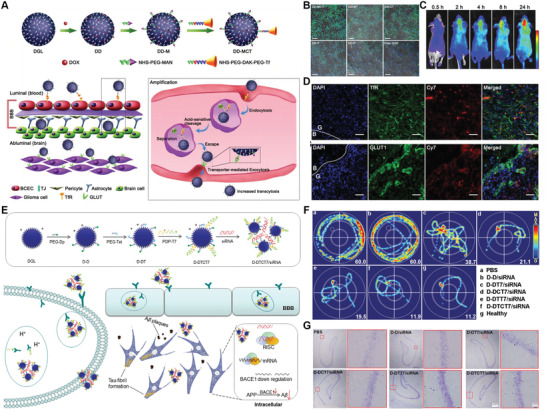
Brain‐targeting NPs with pH‐cleavable linker for accelerating intracellular transport. A) Schematic diagram of preparation of DD‐MCT and proposed mechanism of intracellular pH‐responsive detachment and glioma‐targeting delivery. B) Confocal images of C6 glioma cells in the acceptor chamber of transwell model after incubation with different formulations at donor chamber for 4 h. C) Fluorescence imaging of glioma‐bearing mice injected with Cy7‐labeled DD‐MCT at different time intervals. D) Fluorescence distribution of DC‐MCT at glioma site after intravenous administration for 24 h, glioma slices were immune‐stained with anti‐TfR antibody and anti‐GLUT1 antibody. Reproduced with permission.^[^ ^96^ ^]^ Copyright 2018, Wiley‐VCH. E) Schematic diagram of the acid‐responsive programmed AD‐targeted delivery depot for D‐DTCT7/siRNA. F) The representative swimming paths of mice in MWM test, numbers in the lower right indicate the time spent to reach the platform. G) Nissl staining in the hippocampus of mice in different treatment groups, the red box shows the hippocampal CA1 area. Reproduced with permission.^[^ ^133^ ^]^ Copyright 2020, Wiley‐VCH.

Inspired by this strategy, our group further developed DOX‐loaded DGL with acid‐cleavable Tf coating outside by long‐chain PEG and MAN decorating inside by short‐chain PEG (DD‐MCT) for targeted glioma treatment (**Figure** **3**A).^[^ ^96^ ^]^ Encouragingly, DD‐MCT meets the requirements at each step of the CRITID delivery cascade. After entering into systemic circulation, DD‐MCT could efficiently escape the phagocytosis by MPS and reduce renal excretion because of the PEGylation and optimal particle size (≈30 nm). The outside Tf coating enables DD‐MCT to specifically recognize and bind the TfR on the luminal side of BECs. Following the internalization into BECs , DD‐MCT underwent an acid‐responsive cleavage of Tf within endosome/lysosome, leading to the detachment of MAN‐decorated DOX‐loaded DGL (DD‐M) from the Tf‐TfR interaction complex. The pH‐responsive detachment and subsequent transporter‐mediated exocytosis accelerate the transcytosis. After entering brain parenchyma, the detached DD‐M still possessed the ability to target glioma cells, due to the overexpression of GLUT1 on glioma cells. Moreover, MAN modification could enhance the internalization of DD‐M by glioma cells via RME. Finally, the internalized DD‐M released the DOX under the trigger of acidic pH in endosome/lysosome given to the acid‐cleavable linker. In vitro transwell study demonstrated that C6 glioma cells in the acceptor chamber possessed the highest fluorescent intensity after incubation at donor chamber for 4 h, suggesting the improved BBB transcytosis efficiency (Figure 3B). In vivo fluorescence imaging showed that DD‐MCT could precisely deliver to glioma site after intravenous injection (Figure 3C). Immunofluorescence images showed that the fluorescence of DD‐MCT was poor colocalization with TfR, whereas was good colocalized with GLUT1 in the glioma region, directly confirming that DD‐MCT could undergo a detachment from Tf‐TfR complex and subsequent MAN‐mediated targeting delivery once entering into brain parenchyma (Figure 3D). Importantly, DD‐MCT could efficiently cross the BBB and enter into brain parenchyma in a greater amount, compared to Tf‐coating and MAN‐decorated NPs without acid‐cleavable linker. As a consequence, DD‐MCT could significantly inhibit glioma growth and prolong the survival time of glioma‐bearing mice, validating the efficiency of pH‐cleavable linkage to accelerate BBBtranscytosis.

A recent work was conducted by our group using similar strategy for targeted AD treatment.^[^ ^133^ ^]^ The resulting acid‐cleavable T7‐coated outside and Tet 1‐decorated inside DGL loaded with BACE1 siRNA and D‐amino acid inhibitor (Dp, D‐TLKIVWGKKKC) (D‐DTCT7/siRNA) showed improved BBB transcytosis and diseased neurons targeting efficiency (Figure 3E). Morris water maze (MWM) was used to assess the effect of learning acquisition in AD mice treated with different NPs. Compared with the mice treated with control groups, mice treated with D‐DTCT7/siRNA took the least time to reach the platform, which was similar to that of healthy mice (Figure 3F). Nissl and H&E staining showed nearly no neuronal hypocellularity and nuclear shrinkage in AD mice treated with D‐DTCT7/siRNA (Figure 3G). These results validated the potential of this D‐DTCT7/siRNA to alleviate the neurotoxicity induced by A*β* plaques deposition and p‐tau phosphorylation, leading to significantly improved cognition of AD mice.

#### 3.3.3 Cell Penetrating Peptide (CPP)

CPPs are a family of small amphipathic or cationic polypeptides, typically consisting of 5–30 amino acids, which can efficiently pass through tissue and cell presumably by recognizing negatively charged residues exposed on the cell membrane instead of specific receptors.^[^ ^134^ ^]^ To date, various CPPs have been identified, including trans‐activating (TAT) peptide, penetratin, transportan, toxins, and poly‐arginine.^[^ ^135^ ^]^ Qin et al. modified TAT onto liposomes (TAT‐liposomes) and showed that TAT‐liposomes could enhance the accumulation in brain 2.54‐fold higher compared to unmodified liposomes after intravenous injection.^[^ ^136^ ^]^ However, due to their nonspecific affinity to various cells membrane, CPPs‐mediated brain delivery systems are often characterized with poor brain‐targeting efficiency and high nonspecific distribution after systemic administration.^[^ ^136^ ^]^ Recently, CPP has been explored for combining with peptide‐targeting ligands (e.g., T7 and RGD^[^ ^137^ ^]^), in order to simultaneously address both the issues of brain targeting and BBB transcytosis. Zong et al. developed dual targeting DOX liposomes conjugated with TAT and T7 (DOX‐T7‐TAT‐LIP) for transporting drugs across the BBB and then targeting glioma. In vivo imaging demonstrated that DOX‐T7‐TAT‐LIP possessed higher accumulation at glioma site compared to T7 or TAT‐modified liposomes, suggesting that combination with CPPs could potentially increase the amounts of ligand‐modified NPs reaching brain parenchyma.^[^ ^102^ ^]^ Moreover, tandem peptide with dual‐functionalities has emerged as a promising alternative, comprising a specific peptide in the front position and a CPP in the backend.^[^ ^138^ ^]^ This kind of tandem peptide possesses both specific targeting and high cell penetrating capacity. Liu et al. has constructed a cyclic RGD (RGDfK) and octa‐arginine (R8) (R8‐RGD), in which RGD domain can specifically target integrin *α*_v_ *β*_3_ family and R8 domain can facilitate cell membrane penetration.^[^ ^130^ ^]^ The R8‐RGD was further modified onto PTX‐loaded liposomes (R8‐RGD‐lipo) that could efficiently cross the BBB endothelium and significantly improve the accumulation of PTX at glioma site. As expected, C6 glioma‐bearing mice showed much prolonged median survival time after treatment with R8‐RGD‐lipo compared to R8 or RGD‐modified liposomes separately. All these results indicated that the CPPs have the potential to facilitate intracellular transport of brain‐targeting NPs across the BBB.

### 3.4 Diseased Cell Targeting

**Figure 4:**
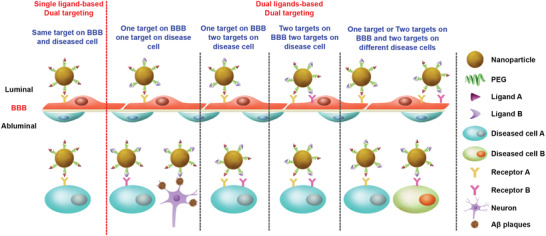
Illustration of dual‐targeting strategies based on single ligand or dual ligands for targeted treatment of brain diseases.

Based on the fact that cells in pathological state often exhibit altered expression profiles, the upregulated molecules of certain diseases have been identified as specific targets of diseased cell population, such as TfR^[^ ^34a^ ^]^ on glioma cells or A*β* plaques^[^ ^36^ ^]^ on neuronal cell from AD. By integrating both the BBB‐specific target and the diseased cells‐specific target, dual‐targeting NPs has been developed to meet this requirement.^[^ ^139^ ^]^ To date, numerous dual‐targeting NP delivery systems have been developed by functionalizing with either single ligand or dual ligands and demonstrated promising BBB‐targeting and diseased cell‐targeting efficiency, which can be categorized into several patterns (**Figure** **4** and **Table** **1**).

**Table 1:** Overview of representative dual‐targeting NPs based on single ligand or dual ligands for targeted treatment of different brain diseases

Disease model	Targeting ligand	Receptor/Transporter	Target site	Carrier	Drug	Ref
Glioma	ApoE peptide	LDLR	BBB/glioma	CP	SAP^a)^	^[^ ^140^ ^]^
Glioma	Angiopep‐2	LRP	BBB/glioma	PCL‐PEG	PTX	^[^ ^141^ ^]^
Glioma	Tf	TfR	BBB/glioma	PAMAM‐G4	DOX	^[^ ^142^ ^]^
Glioma	T7	TfR	BBB/glioma	DGL	DNA	^[^ ^143^ ^]^
Glioma	MAN	GLUT	BBB/glioma	BSA NPs	DOX	^[^ ^144^ ^]^
AD	B6	TfR	BBB/neuron	PLA‐PEG	NAP^b)^	^[^ ^145^ ^]^
AD	RVG29	nAchR	BBB/neuron	DGL	siRNA	^[^ ^146^ ^]^
AD	ApoE	LDLR	BBB/neuron	PS80‐PLGA	Curcumin	^[^ ^147^ ^]^
PD	Angiopep‐2	LRP	BBB/neuron	DGL	hGDNF^c)^	^[^ ^148^ ^]^
PD	RVG29	nAchR	BBB/neuron	Liposome	BPD^d)^	^[^ ^149^ ^]^
Stroke	T7	TfR	BBB/neuron	Mn_3_O_4_@erythrocyte CeO_2_ NP	N/A	^[^ ^150^ ^]^
Stroke	Angiopep‐2	LRP	BBB/neuron		N/A	^[^ ^151^ ^]^
One target on BBB and one target on disease cell						
Glioma	Lf/RGD	LfR/Integrin receptor	BBB/glioma	Fe_3_O_4_/Gd_2_O_3_	Cisplatin	^[^ ^152^ ^]^
AD	TfRaptamer/Tau aptamer	TfR/Tau	BBB/neuron	Circular aptamer	DNA	^[^ ^153^ ^]^
PD	B6/MA^e)^	TfR/DAT	BBB/neuron	Liposome?	EGCG^f)^	^[^ ^101^ ^]^
Stroke	T7/SHp	TfR/GluR	BBB/neuron	Liposome	ZL006^g)^	^[^ ^154^ ^]^
One target on BBB and two targets on diseased cell						
BM	MsTfRmAb/trastuzumab	TfR/HER2	BBB/BM	Polymalic acid	AON^h)^	^[^ ^155^ ^]^
Glioma	Tf/MAN	TfR/GLUT	BBB/glioma	DGL	DOX	^[^ ^96^ ^]^
Two targets on BBB and two targets on diseased cell						
Glioma	MAN/Tf	GLUT1/TfR	BBB/glioma	Liposome	DNR^i)^	^[^ ^156^ ^]^
Glioma	Angiopep‐2/RGD	LRP/Integrin receptor	BBB/glioma	PAMAM‐G5	Gd‐DTPA	^[^ ^157^ ^]^
One target on BBB and two targets on different diseased cells						
Glioma	CDX/RGD	nAchR/Integrin receptor	BBB/NEC^j)^/glioma	Liposome	DOX	^[^ ^158^ ^]^
Two targets on BCEC and two targets on different disease cells						
Glioma	T12/mannose	TfR/SPARC^k)^/MR	BBB/glioma/TAM	BSA NPs	DSF/Cu, Rego^l)^	^[^ ^159^ ^]^
BM	ApoE/iRGD	LDLR/Integrin receptor	BBB/glioma/TAM	Terpolymer‐lipid NPs	DOX/MMC^m)^	^[^ ^160^ ^]^

^a)^ saporin;

^b)^ NAPVSIPQ;

^c)^ the gene encoding human glia cell‐derived neurotrophic factor;

^d)^ N‐3,4‐bis(pivaloyloxy)‐dopamine;

^e)^ mazindol;

^f)^ epigallocatechin gallate;

^g)^ a small molecule neuroprotectant;

^h)^ anti‐sense oligonucleotides;

^i)^ daunorubicin;

^j)^ neovascular endothelial cells;

^k)^ secreted protein acidic and rich in cysteine;

^l)^ kdisulfiram/copper, regorafenib;

^m)^ mitomycin.

#### 3.4.1 Single Ligand‐Based Dual Targeting

**Figure 5:**
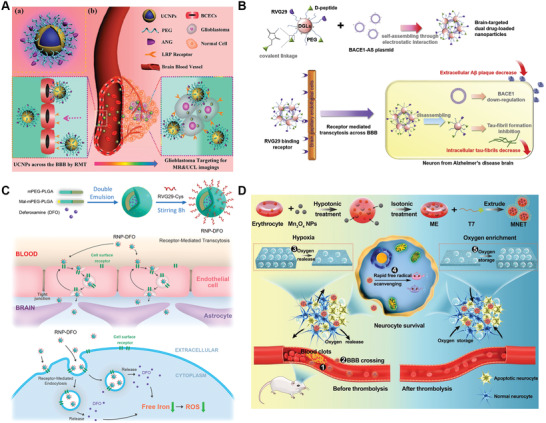
Single ligand‐based dual‐targeting delivery. A) Schematic mechanism of ANG/PEG‐UCNPs for dual‐targeting delivery to glioma cells. Reproduced with permission.^[^ ^161^ ^]^ Copyright 2014, American Chemistry Society. B) Schematic diagram of the preparation and mechanism of DGL‐PEG‐RVG29‐D‐peptide/DNA NPs for dual‐targeting delivery to neurons from AD brain. Reproduced with permission.^[^ ^146^ ^]^ Copyright 2016, Elsevier. C) Schematic diagram of the synthesis and mechanism of RNP‐DFO for dual‐targeting delivery to dopaminergic neurons from PD brain. Reproduced with permission.^[^ ^164^ ^]^ Copyright 2018, American Chemistry Society. D) Schematic diagram of preparation and mechanism of MNET for dual‐targeting delivery to neurons from ischemic stroke brain. Reproduced with permission.^[^ ^150^ ^]^ Copyright 2020, American Chemical Society.

Given that some targets are simultaneously expressed on both the BECs and diseased cell, such as LRP, TfR, or nAchR, dual‐targeting delivery system using single ligand to sequentially recognize the same receptor has been widely explored. For targeting delivery to brain tumor, Ni et al. developed angiopep‐2 functionalized and PEGylated upconversion NPs (ANG/PEG‐UCNPs). ANG/PEG‐UCNPs can sequentially target the LRP on both BBB and glioma cells, leading to precise localization and enhanced accumulation at glioma site (**Figure** **5**A).^[^ ^161^ ^]^ As a consequence, ANG/PEG‐UCNPs functioned as a magnetic imaging/fluorescence imaging agent and showed an improved imaging performance at glioma site compared to clinically used single MRI contrast (Gd‐DTPA) and fluorescent dye (5‐ALA). Our group has also developed several single ligand‐modified brain‐targeting delivery systems with similar dual‐targeting functionality, such as angiopep‐2‐modified AuNPs,^[^ ^92^ ^]^ D‐T7‐modified bilirubin NPs^[^ ^162^ ^]^ and RRGD‐modified AuNPs.^[^ ^163^ ^]^ For AD treatment, Liu et al developed a RVG29‐functionalized DGL loaded with therapeutic gene and peptide (DGL‐PEG‐RVG29‐D‐peptide/DNA NPs) (Figure 5B).^[^ ^146^ ^]^ DGL‐PEG‐RVG29‐D‐peptide/DNA NPs was able to sequentially bind the nAchR expressed on both the BBB and diseased neurons, leading to enhanced accumulation of therapeutic gene and peptide in diseased neurons. After internalization into neuron, the released pBACE1‐AS downregulated BACE1 expression and thus resulted in a reduction of extracellular A*β* plaques. Meanwhile, the D‐peptides could inhibit tau‐fibril formation, exerting a synergistic effect in preventing AD progress. Similar strategy based on RVG‐29‐mediating dual‐targeting delivery has also been used for targeted PD treatment. Qu et al. developed dual‐targeting N‐3,4‐bis(pivaloyloxy)‐dopamine (BPD, a dopamine derivative)‐loaded liposomes functionalized with RVG29 (BPD‐RVG29‐lip) and showed promising dopaminergic neurons targeting efficiency (Figure 5C).^[^ ^149^ ^]^ For targeted stroke therapy, Shi et al. reported an engineered nanosponge by coating Mn_3_O_4_ with erythrocyte membrane and further decorating with T7 peptide (Mn_3_O_4_@nanoerythrocyte‐T7, MENT) (Figure 5D).^[^ ^150^ ^]^ MENT could cross the BBB by TfR‐mediated transcytosis and then preferentially home to diseased neurons by recognizing TfR, acting as a dual‐targeting delivery system. In vivo study demonstrated that treatment with MENT significantly reduced cerebral infarction volume and eliminated free radical compared to control groups, leading to better therapeutic outcomes. All these results validated the effectiveness of single ligand‐based dual‐targeting delivery system in improving targeting efficiency to diseased cells.

#### 3.4.2 Dual Ligands‐Based Dual Targeting

**Figure 6:**
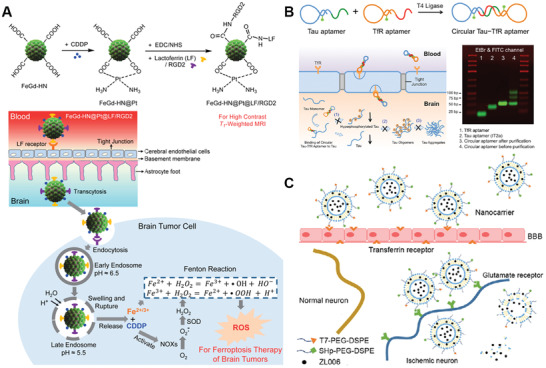
Dual‐targeting strategy based on one target on BBB and one target on diseased cell. A) Schematic mechanism of FeGd‐NH@Pt@LF/RGD2 for dual‐targeting delivery to glioma cells. Reproduced with permission.^[^ ^152^ ^]^ Copyright 2018, American Chemical Society. B) Schematic diagram of preparation and mechanism of circular Tau‐TfR aptamer for dual‐targeting delivery to p‐tau protein from AD brain. Reproduced with permission.^[^ ^153^ ^]^ Copyright 2020, American Chemical Society. C) Schematic mechanism of T7/SHp‐P‐LPs/ZL006 for dual‐targeting delivery ischemic neuron. Reproduced with permission.^[^ ^154^ ^]^ Copyright 2016, Elsevier.

However, the targeting delivery efficiency of single ligand‐functionalized NPs may be affected by several issues, including 1) diseased cells change dynamically with disease progression;^[^ ^165^ ^]^ 2) RMT is a saturable process as the recycling and synthesis of receptors takes time;^[^ ^166^ ^]^ 3) different receptors are often upregulated on diseased cells and upregulation of alternative receptors is involved in drug resistance.^[^ ^167^ ^]^ In contrast, dual targeting NPs using two different ligands may lead to better and controllable diseased cell selectivity. Based on the role of each ligand, dual ligands‐based dual‐targeting delivery system can be further categorized into several types (Figure 4) (**Figure** **4**, **6**).

##### One Target on BBB and One Target on Diseased Cell

Shen et al. developed cisplatin‐loaded Fe_3_O_4_/Gd_2_O_3_ hybrid NPs with conjugation with lactoferrin (Lf) and RGD dimer (RGD2) (FeGd‐NH@Pt@LF/RGD2) for dual‐targeting delivery to brain tumor (Figure 6A).^[^ ^152^ ^]^ FeGd‐NH@Pt@LF/RGD2 NPs was able to target BBB through Lf‐TfR interaction and further target brain tumor cells through RGD2‐integrin *α*_v_ *β*_3_ interaction. Upon endosomal uptake and degradation, FeGd‐NH@Pt@LF/RGD2 could effectively release Fe^2+^, Fe^3+^, and CDDP to exert ferroptosis therapeutic function. In vivo studies demonstrated that FeGd‐NH@Pt@LF/RGD2 NPs could selectively home to brain tumor site with much higher concentration than non‐modified ones, indicating the effectiveness of dual ligands in enhancing brain tumor‐targeting efficiency. Importantly, treatment with FeGd‐NH@Pt@LF/RGD2 NPs led to significant tumor cell growth inhibition compared to single ligand‐modified NPs or non‐modified NPs.

This strategy has also been applied for AD treatment. Li et al. synthesized a circular bifunctional aptamer (Tau‐TfR Aptamer) possessing the dual‐targeting ability for better taupathy therapy (Figure 6B).^[^ ^153^ ^]^ The circular aptamer consists of TfR aptamer that targets TfR on the BBB and tau protein aptamer that selectively targets tau in diseased neuron, thus inhibiting tau phosphorylation and other tauopathy‐related pathological events in the brain. In vivo studies showed that this Tau‐TfR bifunctional aptamer could efficiently disrupt taupathy and significantly improve traumatic brain injury (TBI)‐induced cognitive/memory deficits.

SHp (CLEVSRKNC), a stroke‐homing peptide, is identified and optimized by in vivo phage display in a focal cerebral ischemia rat model.^[^ ^168^ ^]^ Zhao et al. designed a neuroprotectant (ZL006)‐loaded dual‐targeting delivery system based on T7 and SHp co‐modified liposomes (T7/SHp‐P‐LPs/ZL006) for targeting ischemia therapy (Figure 6C).^[^ ^154^ ^]^ Ex vivo fluorescence imaging indicated that DiR labeled T7/SHp‐P‐LPs could efficiently cross the BBB and accumulated selectively in ischemic region rather than cerebral hemisphere of middle cerebral artery occlusion (MCAO) rats. This is mainly attributed to the T7‐mediated BBB targeting and transcytosis and following SHp‐mediated ischemic neuron targeting. Furthermore, MACO rats treated with T7/SHp‐P‐LPs/ZL006 demonstrated improved in vivo anti‐ischemic stroke efficiency compared to T7 or SHp‐functionalized NPs. All these results indicated the potential of dual ligands‐based dual‐targeting delivery system in improving brain‐targeting efficiency and treatment outcome.

##### One Target on BCEC and Two Targets on Diseased Cell

**Figure 7:**
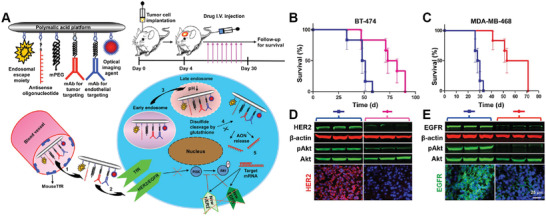
Dual‐targeting strategy based on one target on BBB and two targets on diseased cell. A) Schematic illustration of the functional moieties of dual‐targeting nanoconjugates and proposed mechanism of dual‐targeting delivery to BM after intravenous injection. Survival curve of HER2^+^ BT‐474 BM‐bearing mice treated with B) P/trastuzumab/MsTfR‐mAb/HER2‐AON or C) P/Hu/MsTfR‐mAb/EGFR‐AON. D,E) Western analysis of tumors excised from mouse brains. Reproduced with permission.^[^ ^155^ ^]^ Copyright 2015, American Chemical Society.

The existence of alternative receptor allows for designing dual‐targeting delivery system that can target one receptor on BECs and two receptors on diseased cells. BM developed from a significant proportion of breast cancer is reported to overexpress EGFR‐2 (HER2/neu)^[^ ^169^ ^]^ or from triple‐negative breast cancer (TNBC) expressing EGFR‐1.^[^ ^170^ ^]^ To enable precise targeted BM treatment, Patil et al. developed dual‐targeting nanoimaging agents (NIAs) based on anti‐mouse TfR (MsTfR) mAb and tratuzumab (a mAb target HER2) co‐modified poly(*β*‐_L_‐malic acid) polymer (**Figure** **7**A).^[^ ^155^ ^]^ MsTfR mAb was able to target the BBB and mediate the transcytosis of NIAs across the BBB by recognizing the TfR. After entering into brain parenchyma, NIAs could further target tumor cells mediated by MsTfR mAb‐TfR interaction and trastuzumab‐HER2 interaction synergistically. They also developed EGFR‐targeting NIAs by functionalizing with MsTfR and cetuximab (a mAb target EGFR). In vivo MRI imaging of mice‐bearing EGFR and HER2‐overexpressed double BM demonstrated that the EGFR‐targeting NIAs selectively accumulated at the EGFR‐overexpressing TNBC site only. Similarly, HER2‐targeting NIAs selectively accumulated at the HER2‐overexpressing metastatic breast tumor site, indicating its high targeting specificity. More importantly, after loading with morpholino antisense oligonucleotides (AONs) for inhibition of HER2/neu (HER2‐AON), P/trastuzumab/MsTfR‐mAb/HER2‐AON could inhibit the synthesis of new HER2 and significantly prolong the survival time of HER2^+^ BT‐474‐bearing mice by 57% compared to PBS‐treated mice (Figure 7B,D). Similar benefits were also observed in EGFR^+^ MDA‐MB‐468‐bearing mice treated with P/Hu/MsTfR‐mAb/EGFR‐AON (Figure 7C,E). All the results suggested the high selectivity of the dual‐targeting delivery nanoconjugates for improved BM treatment.

##### Two Targets on BCEC and Two Targets on Diseased Cell

Similarly, the existence of multiple receptors on the BBB endothelium also offers the opportunity to design dual ligands‐modified NPs that target two receptors on BECs and further target two receptors on diseased cells. Yan et al. developed dual‐targeting nanoprobe based on RGD and angiopep‐2 co‐modified PAMAM‐G5 (Den‐RGD‐Angio) for precise diagnosis of brain tumor.^[^ ^157^ ^]^ Den‐RGD‐Angio was designed to first recognize the integrin *α*_v_ *β*_3_ and/or LRP on the BBB endothelium, mediating its transcytosis across the BBB via RMT. After entering into brain parenchyma, Den‐RGD‐Angio could further home to the glioma site via recognizing both overexpressed integrin *α*_v_ *β*_3_ and LRP on glioma cells. In vivo imaging studies showed that Den‐RGD‐Angio not only efficiently crossed the BBB, but also preferentially accumulated at the orthotropic U87MG human glioma xenograft with high selectivity, validating the effectiveness of dual‐targeting delivery system for targeted brain tumor diagnosis.

##### Two Targets on BCEC and Two Targets on Different Diseased Cells

**Figure 8:**
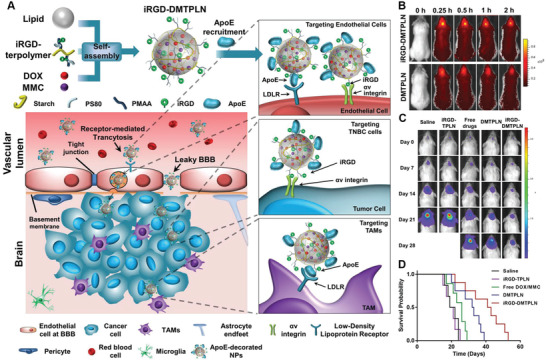
Dual‐targeting strategy based on two targets on BBB and two on different diseased cells. A) Schematic diagram of functional moieties of iRGD‐DMTPLN and proposed mechanism of dual‐targeting delivery to TNBC and TAMs, respectively. B) In vivo fluorescence images of BM‐bearing mice post‐injection with iRGD‐DMTPLN and DMTPLN at different time intervals. C) In vivo bioluminescence images of representative BM over 28 days. D) Survival plot of BM‐bearing mice treated with different formulations. Reproduced with permission.^[^ ^160^ ^]^ Copyright 2019, Wiley‐VCH.

The infiltration of tumor‐associated macrophages (TAMs) is reported to be involved in promoting tumor progression. Therefore, in addition to targeting cancer cell, targeting TAMs in the tumor microenvironment may also be a potential option. Zhang et al. developed a DOX and MMC co‐loaded TPLN modified with iRGD (iRGD‐DMTPLN) for targeted BM treatment (**Figure** **8**A).^[^ ^160^ ^]^ Once entering into systemic circulation, the PS80‐containing iRGD‐DMTPLN could recruit the ApoE onto the surface of NPs to recognize LDLR on the BBB endothelium, leading to BBB transcytosis. Meanwhile, iRGD (CRGDK/RGPD/EC), a cyclic nine amino acid, could further improve targeting efficiency by recognizing integrin *α*_v_ *β*_3_ and enhance the penetration through the exposure of CendR motif to neuropilin‐1 receptor on BECs and tumor cells. Following entrance into brain parenchyma, ApoE‐decorated iRGD‐DMTPLN was able to actively target either LDLR‐expressing TAMs or integrin *α*_v_ *β*_3_‐expressing TNBC in BM, respectively. In vivo imaging showed that iRGD‐TPLN precisely accumulated in BM site with much higher signal than that of unmodified NPs, indicating the ability to selectively target different diseased cells (Figure 8B). In vivo bioluminescence images showed that the combination of DOX and MMC exerted a synergistic effect in inhibiting the TNBC proliferation and reducing the TAMs population (Figure 8C). More importantly, the median survival time of MDA‐MB‐231‐luc‐D3H2LN BM‐bearing mouse treated with iRGD‐DMTPLN was significantly prolonged, indicating the potential of targeting different cell populations in improving BM treatment outcome (Figure 8D).

### 3.5 Diseased Cell Internalization

Generally, the RME can be divided into three stages: first particles sticking to the membrane, second the membrane wrapping the particles and finally the pinch‐off (particle‐lipids complex detaching from the membrane).^[^ ^171^ ^]^ Accumulating evidence indicating that physicochemical properties (such as size,^[^ ^172^ ^]^ shape,^[^ ^49b^ ^]^ and elasticity^[^ ^173^ ^]^) and ligand properties (such as ligand density, ligand length, and rigidity)^[^ ^171b^ ^]^ play a critical role in determining the internalization process via RME. Herein, we mainly focus on how these factors affect RME and provide a guidance for the rational design of brain‐targeting NPs.

#### 3.5.1 Physicochemical Properties of NPs

The cellular internalization of ligand‐modified NPs through RME was widely reported to be strongly dependent on size. Both experiment and theoretical investigations conducted by several groups revealed that the optimal size range of NPs modified with fixed ligand density for efficient uptake is 25–50 nm, while exceedingly large or small NPs would yield inefficient uptake.^[^ ^172^ ^]^ Specifically, Jiang et al. prepared multivalent engineered gold NPs (GNPs) by conjugating multiple molecules to selectively control specific interactions between Herceptin and its receptor HrbB2, a receptor tyrosine kinase overexpressed in various ovarian and breast cancers.^[^ ^172^ ^]^ On the one hand, changes in particle size affect the binding capacity of Her‐GNPs with ErbB2 receptors, with larger NPs possessing higher binding avidity while smaller NPs possessing lower binding avidity. Therefore, larger Her‐GNPs can firmly anchor on cell surfaces while smaller NPs dissociate from the receptors before being engulfed by membrane. On the other hand, however, the internalization process is also highly relevant with membrane wrapping time, the inability of larger NPs to recruit sufficient receptor limit the process of membrane wrapping. By comparing the internalization of the Her‐GNPs in different size, they found that NPs with size ranging from 40–50 nm possessed the most efficient uptake. It should be noted that the endocytic process is a direct attenuation mechanism for membrane receptor presentation. Treatment with 40–50 Her‐GNPs showed the most significant fluctuation with a 40% reduction, suggesting 40–50 nm NPs was the critical cutoff point for RME.

The geometries of the NPs have also attracted much attention because the cell membrane might interact with the NPs in various shapes. To study the effect of shape on the endocytosis rate of NPs, Vacha et al. compared the RME of spherocylinders (cylinder with hemispherical caps) and cylinders using molecular simulations. They found that cylindrical particles (i.e., rodlike particles with no spherical caps) were unable to undergo efficient endocytosis even with a strong ligand‐receptor interaction. Instead, these cylindrical rods remained attached to the membrane, due to the high local curvature. In contrast, it is thermodynamically more favorable to encapsulate a homogeneous spherocylinder of any length than a sphere of the same radius because the mean curvature is only half of the corresponding sphere, while the attractive energy per unit area remains the same.^[^ ^49b^ ^]^

The elasticity also plays an important role in the cellular internalization of NPs. However, conflicting experimental results have been reported in terms of the relation between cellular uptake efficiency and NPs’ elasticity. For instance, Takechi‐Haray et al. reported that liposomes with higher bending rigidity demonstrated a higher internalization by Hela cells than those with lower bending rigidity.^[^ ^174^ ^]^ In another study, Sun et al. found that hyaluronic acid layer‐by‐layer capsules with lower rigidity had a higher cellular uptake than that with higher rigidity.^[^ ^175^ ^]^ By using a coarse‐grained molecular dynamics (CGMD) model, Shen et al. systemically investigated the receptor‐mediated wrapping of elastic NPs with different sizes and shapes. They showed that soft NP was much slower to be fully wrapped than the rigid NP, and their difference in wrapping time increases with the increase in particle size.^[^ ^173^ ^]^ Given to large potential variables, such as NP materials, mechanical properties, cell lines, and receptor expression, it is difficult to make a direct comparison among different experiments and draw a solid conclusion.

#### 3.5.2 Ligand Properties

In addition to particle size, ligand properties present another important factor governing the endocytic kinetics and energetics of ligand‐modified NPs.^[^ ^176^ ^]^ Ding et al. applied the dissipative particle dynamics to systemically study the effect of ligand properties (e.g., ligand density, length, and rigidity) on the RME. They found that the NP decorated with longer ligands was more likely to attach to the membrane, while it was harder to be totally engulfed. Increasing the ligand density and rigidity, which enhances the uniform distribution of ligands on the NPs, may lead to the total engulfment. They also investigated the effect of the hydrophobicity/lipophobicity of ligands on the RME and reported that ligand‐modified NPs cannot be totally engulfed when the ligand was totally hydrophilic. By contrast, NPs can be totally engulfed by the membrane when coating with amphiphilic ligand without the help of lipids.^[^ ^171b^ ^]^ Moreover, high ligand density may increase cellular internalization efficiency of NPs but also compromise the stealth surface of NPs during systemic circulation. Thus, rationally designing brain‐targeting NPs with optimized ligand properties to ensure both precise targeting and effective diseased cell internalization is of great importance.

### 3.6 Intracellular Drug Release

Recently, great effort has been devoted to the development of intracellular stimulus‐responsive drug release system that are able to stay stable under extracellular condition while to trigger rapid drug release in response to intracellular stimuli (e.g. acidic pH, redox, reactive oxygen species) following internalization present the most popular strategy.^[^ ^177^ ^]^ Such intracellular stimulus‐responsive drug release ensures drug release at the target site (spatial control) and/or at the right time (temporal control), leading to a better bioavailability while reduced site‐effect. To enable stimulus‐responsive drug release property, drugs are usually required to either undergo appropriate modification to conjugated onto NPs delivery system via stimulus‐responsive linker or encapsulate into stimulus‐responsive NPs. Moreover, drug with chemical modification (referring as prodrug) is beneficial for not only controlling over intracellular drug release but also for improving pharmacokinetic and pharmacodynamic performance. However, it is worth noting that drug modification should not cause significant loss of efficacy of original drugs or change to pharmacological properties post‐release, such as structure, physicochemical properties, therapeutic target or mechanism, and biological activity. Herein, we review and highlight the current state‐of‐art stimulus‐responsive strategies integrated into the design of brain‐targeting delivery system.

#### 3.6.1 Endo/lysosomal pH‐Responsive Drug Release

**Figure 9:**
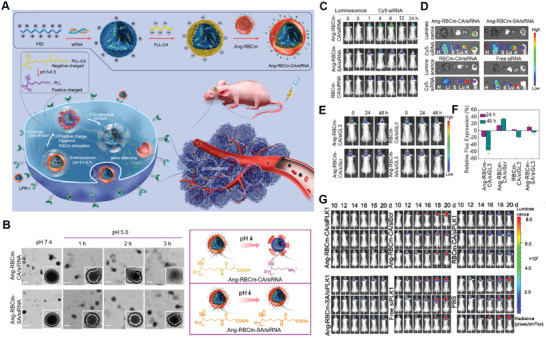
Biomimetic brain‐targeting siRNA delivery system with pH‐responsive release capacity. A) Schematic diagram of fabrication and proposed mechanism of Ang‐RBCm‐CA/siRNA. B) TEM images of Ang‐RBCm‐CA/siRNA and Ang‐RBCm‐SA/siRNA incubated at pH 7.4 or 5.0 for 1, 2, or 3 h. Scale bar: 200 nm. Illustration of the mechanism pH‐triggered Ang‐RBCm‐CA/siRNA membrane disruption. C,D) In vivo and ex vivo fluorescence of orthotopic U87MG‐luc human glioma‐bearing nude mice post‐injection with different formulations at different time intervals. E) Luciferase expression of the brain in mice before or post‐injection of different formulations. F) Semi‐quantitative analysis of the bioluminescence intensity. G) Luminescence images of orthotopic U87MG‐luc human glioma‐bearing nude mice treated with different siPLK1‐loading formulations. Reproduced with permission.^[^ ^179^ ^]^ Copyright 2020, American Chemical Society.

It is well‐known that pH variations exist between different intracellular compartments. For instance, the pH in cytosol, similar to normal tissue and blood, is around 7.4, while the pH decreases in endosome (5.5–6.8) and in lysosome (4.0–5.0).^[^ ^177^, ^178^ ^]^ Given that the intrinsic pH gradient, brain‐targeting NPs with pH‐responsiveness may hold great promise to release drug in specific intracellular compartments. Generally, pH‐responsive NPs are designed to be able to deform (by swelling, dissolution, or collapse) or cleave pH‐labile linker under endo/lysosomal pH conditions, resulting in efficient intracellular drug release. Liu et al. developed a novel charge conversional biomimetic siRNA nanoplatform for targeted RNAi therapy (**Figure** **9**A).^[^ ^179^ ^]^ In order to realize intracellular siRNA release, an endo/lysosome pH triggered charge‐conversion strategy which reverses the charge from negative to positive was adopted by conjugating a pH‐sensitive citraconic anhydride (CA). The pH‐responsive charge reversion in turn triggered the release of siRNA and accelerated RBC membrane disruption. Moreover, the released siRNA could further escape from lysosomal to elicit cytoplasmic RNAi machinery. TEM images demonstrated a complete structural collapse of Ang‐RBCm‐CA/siRNA in a mild acid environment (pH 5.0). In contrast, the structure of Ang‐RBCm‐SA/siRNA containing pH‐stable succinic anhydride remained intact in the same condition (Figure 9B). In vivo and ex vivo fluorescence imaging demonstrated the excellent targeting ability of Ang‐RBCm‐CA/siRNA to glioma site compared to unmodified one (Figure 9C,D). Moreover, luciferase expression of brain in mice demonstrated a stronger reduction of glioma bioluminescence after treatment with Ang‐RBCm‐CA/siGL3 than that treated with control formulations (Figure 9E). Semi‐quantitative analysis showed that Ang‐RBCm‐CA/siGL3 induced 16% and 58% bioluminescence reduction (relative to initial bioluminescence intensity) in the glioma at 24 and 48 h post‐injection (Figure 9F). Most importantly, in vivo bioluminescence imaging demonstrated a significant lower intensity after treatment with Ang‐RBCm‐CA/siPLK1 (therapeutic siRNA) than non‐targeted one with pH‐responsiveness as well as targeted one without pH‐sensitivity (Figure 9G).

Our group has synthesized a pH‐sensitive DOX probe containing an acid‐labile hydrozone bond.^[^ ^92^ ^]^ The pH‐sensitive DOX probe was able to conjugate onto AuNPs through “S—Au” chelation to form DOX‐loaded AuNPs, wherein the hydrozone could be cleaved in response to acid pH condition in aqueous solution. In vitro studies demonstrated that the pH‐sensitive DOX could be released effectively from AuNPs in a pH‐dependent and time‐dependent manner. In vivo studies validated that DOX could also be released efficiently in glioma site whereas rarely in normal tissues, resulting in improved chemotherapeutic effect and reduced side‐effects.^[^ ^92^, ^180^ ^]^ As described in Section 3.3.2, similar pH‐responsive release behavior of DOX was also demonstrated by using a pH‐labile imine bond.^[^ ^181^ ^]^ In vitro and in vivo studies suggested that DOX could be released efficiently from DGL under the trigger of acidic pH condition. All these results indicated the pH‐responsive intracellular drug release may be beneficial for improving therapeutic effect while reducing systemic toxicity.

#### 3.6.2 Redox‐Responsive Drug Release

**Figure 10:**
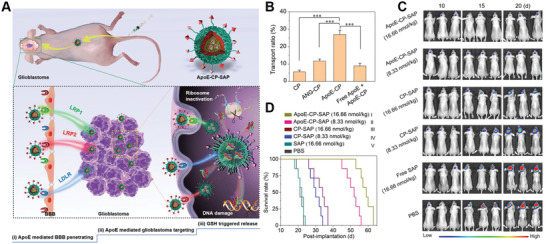
Brain‐targeting delivery system with GSH‐responsive release capacity. A) Schematic mechanism of ApoE‐CP for BBB transcytosis, glioma targeting, and GSH‐triggered SAP release. B) In vitro BBB model transport ratio of Cy5‐labeled CP, ANG‐CP, and ApoE‐CP following 24 h of incubation. Blockade experiments were conducted by pretreating U87 MG cells with free ApoE (100 µg mL^−1^, 30 min). C) In vivo luminescence images of human U87‐MG‐luc glioma‐bearing mice model treated with different formulations (*n* = 7). D) Survival plot of U87‐MG glioma‐bearing mice treated with different formulations. Reproduced with permission.^[^ ^140^ ^]^ Copyright 2018, American Chemical Society.

Redox potential has emerged as an attracting and powerful stimulus for active intracellular drug release.^[^ ^182^ ^]^ It is well‐known that the cellular redox environment is mainly regulated by the level of glutathione (GSH). GSH, a *γ*‐glutamyl‐cysteinyl‐glycine, is the most abundant low‐molecular weight reducing agent present in the cytoplasm of mammalian cells with a critical role in maintaining the cellular oxidative balance. The intracellular concentration of GSH is as high as 2–10 mm that is kept reduced by nicotinamide adenine dinucleotide phosphate (NADPH) and GSH reductase. In sharp contrast, GSH is present at extremely low concentrations of ≈2–20 µm in the body fluid (e.g., blood) or normal extracellular matrices.^[^ ^183^ ^]^ Such a distinct redox gradient from extracellular environment to intracellular cytosol can be leveraged as stimulus for triggering intracellular drug release. Jiang et al. developed an ApoE peptide (LRKLRKRLL)_2_C‐decorated chimeric polymersomes (CP) (ApoE‐CP) for targeting delivery of therapeutic protein to glioma (**Figure** **10**A).^[^ ^140^ ^]^ ApoE peptide, a tandem dimer sequence of the receptor‐binding domain of ApoE, was reported to possess high affinity to multiple LDLR members, such as LDLR, LRP‐1, and LRP‐2 without interfering with endogenous ApoE. ApoE‐CP could specifically bind to LDLRs and mediate superb BBB crossing and further target the glioma cell, functioning as a dual‐targeting delivery system. After internalization into glioma cells, SAP could be released from ApoE‐CP in response to the high GSH concentration, leading to the DNA damage. In vitro BBB model study showed 2.2‐fold higher penetration through the bEnd.3 monolayer than angiopep‐2 decorated CP (Figure 10B). Treatment with SAP‐loaded ApoE‐CP efficiently inhibited the growth of U87 glioma without eliciting any severe adverse effect and significantly improved survival benefits (Figure 10C,D). The results suggested that brain‐targeting delivery system with redox‐responsive drug release hold promise to further improve treatment outcome of brain diseases.

#### 3.6.3 Reactive oxygen species (ROS)‐Responsive Drug Release

**Figure 11:**
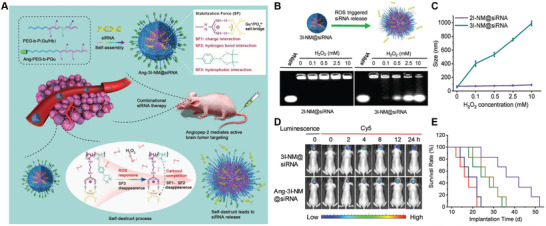
Brain‐targeting delivery system with ROS‐responsive release capacity. A) Schematic diagram of fabrication and proposed mechanism of Ang‐3I‐NM@siRNA for glioma‐targeting delivery and ROS‐triggered siRNA release. B) Schematic illustration of siRNA released from 3I‐NM@siRNA under H_2_O_2_ triggering and gel electrophoresis analysis of siRNA release from 3I‐NM@siRNA following H_2_O_2_ treatment. C) Size change of 3I‐NM@siRNA following H_2_O_2_ treatment determined by DLS. D) Fluorescence imaging of orthotopic U87MG‐luc human glioma‐bearing nude mice at different time points post‐injection of Ang‐3I‐NM@siRNA and 3I‐NM@siRNA. E) Survival plot of glioma‐bearing mice treated with different formulations. Reproduced with permission.^[^ ^186^ ^]^ Copyright 2018, Wiley‐VCH.

ROS is mainly involved in the signal transduction and metabolism. Elevated ROS in cells or tissues, however, often leads to oxidation stress that has implication in a series of diseases including brain tumor and neurodegenerative disease.^[^ ^184^ ^]^ ROS are generally referred to a class of oxygen‐derived chemical species, including hydrogen peroxide (H_2_O_2_), single oxygen (^1^O_2_), superoxide (O_2_^−^) and hydroxyl radicals (HO·).^[^ ^185^ ^]^ Thus, ROS‐responsive drug release may also hold great promise to amplify the treatment outcome of brain‐targeting delivery system. Zheng et al. developed angiopep‐2 modified polymeric siRNA nanomedicine (Ang‐3I‐NM@siRNA) for targeted RNAi therapy (**Figure** **11**A).^[^ ^186^ ^]^ In addition to the improved BBB transcytosis and glioma‐targeting delivery, the developed Ang‐3I‐NM@siRNA demonstrated effective site‐specific release of siRNA, due to the intratumoral ROS‐triggered sequential destabilization. 3I‐NM@siRNA exhibited an active ROS response and efficient siRNA release upon treatment with H_2_O_2_ at concentration above 0.1 × 10^−3^ m, whereas 2I‐NM@siRNA was hard to be released (Figure 11B). In line with gel electrophoresis, dynamic light scattering (DLS) analysis showed a size increase of 3I‐NM@siRNA correlated with the H_2_O_2_ concentration, verifying the ROS‐responsive swelling (Figure 11C). In vivo fluorescence imaging demonstrated a significantly stronger accumulation of Ang‐3I‐NM@siRNA at glioma site at 2 h post‐injection than that of unmodified one (3I‐NM@siRNA), and maintaining a high‐level fluorescence up to 24 h (Figure 11D). Survival curves showed that treatment with Ang‐3I‐NM@(siPLK1+siVEGFR2) markedly increased survival with a median survival time of 36 days, which was significantly longer than mice treated with 3I‐NM@(siPLK1+siVEGFR2) (18 days), Ang‐3I‐NM@(siPLK1) (24 days) and Ang‐3I‐NM@(siVEGFR2) (26 days) (Figure 11E). All these results validated the effectiveness of brain‐targeting delivery combined with ROS‐responsive release in RNAi possessed improved treatment benefit to glioma.

#### 3.6.4 Dual or Multi‐Stimuli‐Responsive Drug Release

It should be noted that the pathological condition of a diseased cell is a complex collection of different heterogeneities. To further fine‐tune drug release performance (versatility, specificity, and sensitivity) and improve therapeutic efficacy, the combination of two or more different stimuli described above can be rationally chosen, such as pH/redox, pH/ROS. Moreover, the triggered drug release under dual or multiple stimuli may occur either simultaneously within diseased cell or in a sequential manner in different intracellular compartments.^[^ ^187^ ^]^ Thus, rationally designing the brain‐targeting delivery system with dual or multi‐stimuli‐triggered release ability may obtain more controllable treatment outcome.

**Figure 12:**
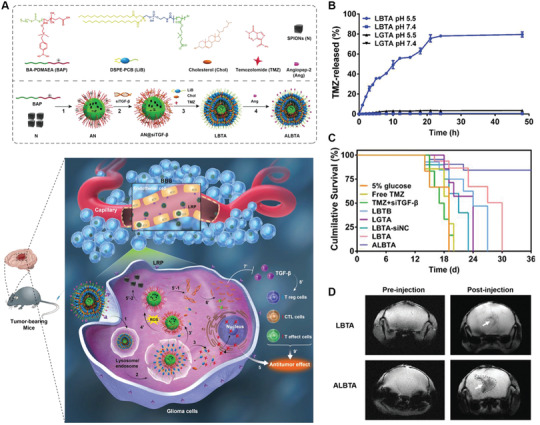
Brain‐targeting delivery system with dual stimuli‐responsive release capacity. A) Schematic diagram of fabrication and proposed mechanism of ALBTA for glioma‐targeting delivery and pH and ROS‐triggered drug release. B) The cumulative release ratio of TMZ from LGTA (blank line) and LBTA (blue line) incubated at pH 7.4 and 5.5 at 37 °C. C) Survival plot of glioma‐bearing mice treated with various formulations. D) Representative in vivo T2‐weighted MRI images of brain in intracranial glioma‐bearing mice before and after injection of LBTA (upper) or ALBTA (lower). Reproduced with permission.^[^ ^189^ ^]^ Copyright 2018, Wiley‐VCH.

Down‐regulation of transforming growth factor‐*β* (TGF‐*β*) has been reported as a promising approach to sensitize the temozolomide (TMZ)‐based chemotherapy via relief of immunosuppressive glioma microenvironment.^[^ ^188^ ^]^ Inspired by this, Qiao et al. developed a dual‐targeting nanotheranostic system (Angiopep lipoPCB (TMZ+BAP/siTGF‐*β*), ALBTA) with pH‐responsiveness and ROS‐responsiveness for targeted intracranial glioma treatment (**Figure** **12**A).^[^ ^189^ ^]^ The proposed ALBTA was able to effectively cross the BBB and accumulated at glioma site selectively. Following the internalization into glioma cells via LRP‐mediated endocytosis, ALBTA was trafficking to endosome/lysosomes: 1) the zwitterionic lipid DSPE‐PCB underwent a charge reversion from negative to positive in response to the acidic pH microenvironment, leading to the perturbation of membrane of endosome/lysosome; 2) TMZ and AN@siTGF‐*β* NPs were more likely to escape into cytoplasm, and the released TMZ further entered into nuclei to kill the glioma cells; 3) meanwhile, AN@siTGF‐*β* could release siTGF‐*β* under the trigger of ROS due to the ROS‐responsive poly[(2‐acryloyl)ethyl(p‐boronic acid benzyl)diethylammonium bromide] (BA‐PDEAEA, BAP) composition; 4) consequently, the downregulation of TGF‐*β* modulated the glioma immunosuppressive microenvironment and eventually exerted a synergistic anti‐tumor effect; In vitro release study showed that only 0.52% TMZ release from LBTA at pH 7.4 after 2 days incubation. In contrast, the cumulative release of TMZ from LBTA at pH 5.5 was increased to 79.57%, indicating that NPs was disrupted due to the protonation of DSPE‐PCB in acidic pH condition, while stable in normal culture condition (Figure 12B). More importantly, this nanosystem significantly prolonged the survival time of glioma‐bearing mice from 19 days (untreated group) to 36 days, validating that chemotherapy and immunotherapy could synergistically inhibit glioma cell proliferation (Figure 12C). Given the intrinsic contrast feature of SPIONs, in vivo MRI images showed an obvious contrast enhancement in mice treated with ALBTA than that treated with LBTA, indicating the efficient targeting and precise trace of ALBTA in brain tumors (Figure 12D). In conclusion, the proposed brain‐targeting nanoplatform with dual‐stimuli responsiveness holds great promise to improve brain tumors treatment outcome.

## 4 Summary and Outlook

The development, evaluation, and revolution of brain targeting delivery system for brain disease treatment have underwent intensive exploration and are still in progress. However, researcher should rethink the designing requirement of brain‐targeting drug delivery system, such as brain‐targeting delivery and BBB transcytosis. This review provided a comprehensive overview on the delivery process of a systemically administrated brain‐targeting delivery system and first proposed a six‐step CRITID delivery cascade. In an effort to guarantee the maximum therapeutic efficacy, rational design of brain‐targeting delivery system should be aware of the issues and/or requirements at each step as well as corresponding strategies for troubleshooting, as summarized in **Table** **2**. The CRITID delivery cascade can serve as a guideline for designing more efficient and specific brain‐targeting delivery system toward diverse brain diseases. Although the proposed CRITID cascades are somehow focusing on ligand‐functionalized brain‐targeting delivery system, the application of the proposed CRITID delivery cascade in other state‐of‐art brain delivery strategies can also be pursued, such as protein corona‐assisted targeting, transient BBB opening and exosomes‐based brain‐targeting delivery. Moreover, brain‐targeting nanoparticles that meet the requirements of CRITID delivery cascade are often characterized with multifunctionality, which might pose a challenge to the scale‐up of manufacturing or clinical translation. Therefore, the balance between multifunctionality and the manufacturing or clinical translation should also be taken into consideration.

**Table 2:** Summary of the issues and/or requirements as well as corresponding troubleshooting strategies in each step of CRITID delivery cascade

Delivery cascad	Issues and/or requirements	Strategies for troubleshooting
Blood circulation	Rapid opsonization, MPS capture, and renal excretion can result in non‐specific distribution and rapid blood clearance. Thus, reducing non‐specific distribution and prolonging blood circulation time of brain‐targeting NPs is required.	Optimize physicochemical properties (size, shape, charge); Surface modification: PEGylation; Self‐peptide (CD47) functionalization; Biomimetic membrane coating (e.g., RBC, leukocytes, platelets, macrophage, cancer cells).
BBB recognition	NPs with the ability to recognize receptors or transporters on the BBB is required.	Functionalizing with targeting ligands or antibodies Coating with immune and tumor cell membrane. CAR‐T cell‐based targeting
Intracellular Transport	High‐affinity ligand‐functionalized NPs may trafficking to lysosome instead of transcytosis, leading to trapping within BECs. Thus, minimizing the intracellular trapping of NPs and accelerating transcytosis is required.	Optimizing ligand affinity or ligand density; Utilizing pH‐dependent antibody or cleavable linker to enable detachment; Cell penetrating peptide
Diseased cell targeting	Brain‐targeting NPs are required to distinguish between diseased cells and normal brain cells after entering brain parenchyma	Dual‐targeting to receptor on BBB and receptor on diseased cell sequentially by functionalizing either single ligand or dual ligands.
Diseased cell internalization	Efficient translocation across the cell membrane to subcellular compartments is required for exerting therapeutic effect if therapeutic targets are localized in intracellular compartments.	Optimizing physicochemical properties of NPs (e.g., size, shape, and elasticity) and ligand properties (e.g., ligand density, ligand length, ligand rigidity).
Intracellular drug release	Drugs are required to be released rapidly in the form of free drugs from NPs and specifically in subcellular compartments, escape lysosomes if their therapeutic targets are located in other compartments, such as mitochondria, nucleus.	Single stimulus‐responsive drug release (e.g., pH‐responsive release, GSH‐responsive release, ROS‐responsive release). Dual stimuli‐responsive drug release (e.g., pH+ROS; pH+GSH).

Despite of the stealth design features, the absorption of protein onto brain‐targeting NPs’ surface during blood circulation seems inevitable. The formation of protein corona may cover the ligands and thus present an obstacle to receptor recognition on the BBB, leading to unsatisfied targeting efficiency and non‐specific biodistribution. However, accumulating evidence suggested that the protein corona, in some case, is rather beneficial. There are numerous functional proteins absorbing on the surface of NPs delivery system that is able to mediate brain‐targeting delivery and BBB transcytosis.^[^ ^190^ ^]^ Thus, protein corona‐modified NPs may present a new impetus for brain‐targeting delivery by selectively manipulating corona composition. Zhang et al. developed a kind of bioinspired liposomes (SP‐slip) by modifying liposomal surface with a short nontoxic peptide derived from A*β*_1–42_ that specifically interacts with the lipid‐binding domain of exchangeable Apos.^[^ ^191^ ^]^ SP‐slip absorb plasma Apo A1, E, and J, consequently exposing receptor‐binding domain of Apos to achieve brain‐targeted delivery. This strategy may provide a thinking to explore the potential of protein corona for developing brain‐targeting NPs meeting the requirements of CRITID delivery cascade.

It has been reported that compromised BBB integrity is associated with the pathogenesis of neurodegenerative disorders and brain tumors, leading to the enlargement of gap between endothelial cells.^[^ ^192^ ^]^ The disrupted BBB in pathological condition creates an opportunity for NPs to enter the brain parenchyma via paracellular extravasation or so‐called enhanced permeability and retention (EPR) effect. However, the limited gap distance in pathological condition allows only a very small fraction of NPs entering into brain parenchyma, which restricts their therapeutic potential. Therefore, transient and reversible BBB opening may represent a promising strategy to further increase the amount of NPs entering into parenchyma without inducing clinically significant worsening.^[^ ^193^ ^]^ Han et al. developed an “autocatalytic” brain tumor‐targeting delivery strategy for readily crossing the BBB and preferentially accumulate at tumor site.^[^ ^194^ ^]^ In this strategy, a small fraction of NPs entered the brain parenchyma through transcytosis or through the BBB gaps. After reaching tumor microenvironment, NPs locally released lexiscan, a small molecule agonist of A2A adenosine receptors, known to reduce the tightness of tight junction and thus transiently enhanced BBB permeability.^[^ ^195^ ^]^ The lexiscan‐induced transient BBB opening allowed more NPs to enter the same region, creating a positive feedback loop for increased delivery. As a consequence, a 94.0‐fold higher accumulation was achieved in the tumor regions than that in non‐tumor regions of the same brain. Similarly, the combination with transient BBB opening with ligand‐modified NPs meeting the requirements of CRITID delivery cascade may further boost their therapeutic potential. In addition to chemical agents, magnetic resonance‐guided focused ultrasound (MRgFUS) coupled with injected microbubbles is emerging as a noninvasive technique to transiently open the BBB in targeted CNS regions.^[^ ^196^ ^]^ Focused ultrasound has been widely used in not only animal model of diseases including brain tumors and neurodegenerative diseases but also human with amyotrophic lateral sclerosis,^[^ ^197^ ^]^ wherein the delivery of a range of therapeutic substrates has been enhanced, including antibodies,^[^ ^198^ ^]^ chemotherapies^[^ ^199^ ^]^ as well as brain‐targeting NPs.^[^ ^200^ ^]^

To date, great successes have been made in the use of synthetic NP‐based brain‐targeting delivery systems for targeted therapy of brain diseases. However, their biotoxicity and immunogenicity remain the biggest challenge to clinical application. The exploration of bioinspired nanocarrier as alternative delivery system has gained increasing interest. Exosomes are a sub‐group of lipid bilayer extracellular vesicles virtually secreted by almost all types of mammalian cells.^[^ ^201^ ^]^ Because of their bioactive cargo, including proteins, coding and noncoding RNAs, and lipids, they are reported to play an important role in intercellular communication by transferring or exchanging their bioactive cargo between parent cells and recipient cells.^[^ ^202^ ^]^ Encouragingly, exosomes have emerged as a promising tool for brain‐targeting delivery owing to their intrinsic features, such as unique structure, biocompatibility, low immunogenicity, colloidal stability, stealth capacity, ability to cross BBB, and cell‐homing capacity.^[^ ^203^ ^]^ The use of exosomes for brain‐targeting delivery of various therapeutic agents has been explored in the treatment of brain diseases, such as tumor^[^ ^204^ ^]^ and AD.^[^ ^205^ ^]^ Furthermore, better targeting efficiency of exosomes can be presumed by engineering surface with ligands, such as c(RGDyK)^[^ ^206^ ^]^ and RVG‐29.^[^ ^207^ ^]^ Therefore, rational design of exosome‐based brain‐targeting delivery system that meets the requirements of CRITID delivery cascade may increase the chance of clinical translation.

Another concern regarding the scale‐up of manufacturing and bench‐to‐bed side clinical translation of these brain‐targeting NPs comes from their multifunctionalities. In fact, none of these actively targeting NPs have advanced past clinical trials until now, even for those toward solid tumors. On the one hand, their relatively complex design complicates the scale‐up of manufacturing process and thus poses a major challenge to the clinical translation. On the other hand, although these have not been the foremost restricting factors in clinical translation of brain‐targeting NPs, more quality control steps, potential neurotoxicity, increased cost, and time consuming remain the predominant concerns when advancing to clinical trials. Moreover, the plethora of factors contributing to the targeting ability of these brain‐targeting NPs makes these approaches hardly predictable. Therefore, to improve the probability of clinical translation of brain‐targeting NPs with, several critical prerequisites should be met in the future: 1) simplify the design as much as possible while still reserve the multifunctionalities that meet the requirements of CRITID delivery cascade; 2) utilize biodegradable and biocompatible materials with low neurotoxicity and cytotoxicity, and these materials are able to be easily eliminated from brain as well; 3) the factors that influence both in vitro and in vivo behavior of NPs should be well documented and evaluated; 4) development of better and more predictive preclinical animal models that are able to well predict the targeting efficiency toward diverse brain diseases; 5) establish good manufacturing practice guidelines and good laboratory practice regulations to bridge the bench‐bed gap.

## Conflict of Interest

The authors declare no conflict of interest.
